# Supplementary material for: Integrative Mendelian randomization for detecting exposure-by-group interactions using group-specific and combined summary statistics
Source: PLoS Genet. 2025 Sep 11;21(9):e1011819. doi: 10.1371/journal.pgen.1011819 (PMC12440225; doi:10.1371/journal.pgen.1011819)
Supplement: S1 Text — This document presents a comprehensive description of our methodological framework, including an extended discussion of the Bayesian hierarchical model and rigorous justification of all hyperparameter choices. It further details the simulation design and the procedures employed for generating summary statistics. S1 Text reports additional simulation results and expands upon the data-analysis findings presented in the main text. (PDF) [file pgen.1011819.s001.pdf]

Supporting Information

Integrative Mendelian randomization for detecting exposure-by-group interactions using group-specific and combined summary statistics

Ke Xu[1,2], Nathaniel Maydanchik[1], Bowei Kang[1], Jianhai Chen[1], Qixiang Chen[1], Gongyao Xu[1], Shinya Tasaki[3], David A. Bennett[3], Lin S. Chen[1,*]

1 Department of Public Health Sciences, The University of Chicago, Chicago, IL, USA

2 Department of Applied and Computational Mathematics and Statistics, University of Notre Dame, IN, USA

3 Department of Neurological Sciences & Rush Alzheimer's Disease Center, Rush University Medical Center, Chicago, IL, USA

* Correspondence should be addressed to Lin S. Chen, lchen4@bsd.uchicago.edu.

S1 Model details

S1.1 Modeling IV-to-outcome effect with unmeasured individual group label

Modeling IV-to-outcome effect in individual level data. Let X denote the risk factor and Y the outcome. At the individual level, we assume the following structural equations:

$$X \mid G, S, U = \sum_{j=1}^{p} \gamma_j G_j + \beta_{U_X} \cdot U + \varepsilon_X \tag{S1}$$

$$Y \mid X, S, G, U = \beta X + \sum_{j=1}^{p} \alpha_j G_j + \beta_{U_Y} U + \beta_{SY} \cdot S + \beta_{\text{int}} \cdot X \circ S + \varepsilon_Y. \tag{S2}$$

In Eqs S1 S2, the vector $G = (G_1, G_2, \ldots, G_p)$ contains p genetic variants for an individual, and $\alpha = (\alpha_1, \ldots, \alpha_p)$ represents the uncorrelated pleiotropy effects. Here, S denotes a binary group label taking values in $\{0, 1\}$, and U is an unmeasured confounder. The parameters of interest, β and β_{int}, capture the causal effect of exposure X on outcome Y and the interaction effect between X and group label S, respectively.

Substituting the IV-to-exposure model Eq S1 into the exposure-to-outcome equation Eq S2 yields

the following individual-level IV-to-outcome effect model:

$$Y \mid S, G, U = \widetilde{c}_0 + \sum_{j=1}^{p} (\beta \cdot \gamma_j + \alpha_j) \cdot G_j + \sum_{j=1}^{p} \beta_{\text{int}} \cdot S \cdot G_j + \widetilde{c}_1 \cdot U + \widetilde{\varepsilon}_Y, \tag{S3}$$

where $\widetilde{c}_0$ is a constant depending on the group indicator S, $\widetilde{c}_1$ is a constant representing the strength of confounding effect. Here, $\widetilde{c}_0$ and $\widetilde{c}_1$ absorb the non-genetic contributions, and $\widetilde{\varepsilon}_Y$ remains a zero-mean error term. Hence, taking the conditional expectation of both sidess yields:

$$\mathbb{E}\left(Y \mid S, G, U\right) = \widetilde{c}_0 + \sum_{j=1}^{p} (\beta \cdot \gamma_j + \alpha_j) \cdot G_j + \sum_{j=1}^{p} \beta_{\text{int}} \cdot S \circ G_j + \widetilde{c}_1 \cdot U. \tag{S4}$$

Modeling interaction effect when individual-level group label not available. When we have GWAS summary statistics but do not have individual-level data, we do not know the individual-level values for the group variable S. We further assume that the group label S follows a Bernoulli distribution (with a proportion parameter ρ), independently of G and U. We have

$$S \sim \text{Bernoulli}(\rho), \quad \rho = \mathbb{E}[S].$$

Based on equation Eq S4, we have

$$\begin{aligned}
\mathbb{E}\left(Y \mid G, U\right) =& \mathbb{E}_S\left(\mathbb{E}(Y \mid S, G, U)\right) \\
=& \mathbb{E}\left(Y \mid S = 0, G, U\right) \cdot P(S = 0) + \mathbb{E}\left(Y \mid S = 1, G, U\right) \cdot P(S = 1) \\
=& \left[\widetilde{c}_0 + \sum_{j=1}^{p} (\beta \cdot \gamma_j + \alpha_j) \cdot G_j + \widetilde{c}_1 \cdot U\right] \cdot (1 - \rho) \\
&+ \left[\widetilde{c}_0 + \sum_{j=1}^{p} \left[(\beta + \beta_{\text{int}}) \cdot \gamma_j + \alpha_j\right] \cdot G_j + \widetilde{c}_1 \cdot U\right] \cdot \rho \\
=& \widetilde{c}_0 + \sum_{j=1}^{p} \left[(\beta + \beta_{\text{int}} \cdot \rho)\, \gamma_j + \alpha_j\right] \cdot G_j + \widetilde{c}_1 \cdot U,
\end{aligned}$$

where $\mathbb{E}_S\left(\cdot\right)$ denotes taking the expectation over the random variable S.

Therefore, the IV-to-outcome structural model becomes

$$Y \mid G, U = \mathbb{E}\left(Y \mid G, U\right) + \widetilde{\varepsilon}_Y = \widetilde{c}_0 + \sum_{j=1}^{p} \left[(\beta + \beta_{\text{int}} \cdot \rho)\, \gamma_j + \alpha_j\right] \cdot G_j + \widetilde{c}_1 \cdot U + \widetilde{\varepsilon}_Y, \tag{S5}$$

where $\widetilde{\varepsilon}_Y$ is a noise term with mean zero.

S1.2 Model identifiability

In the int2MR framework, because the three GWAS datasets use different proportions of comparison-group individuals, we can analytically identify the causal parameters β and β_{int}. Specifically, consider three studies indexed by $k \in \{0, 1, 2\}$, where each study provides marginal SNP-outcome associations, denoted as $\Gamma_{k,j}$ for the j-th instrumental variable (IV).

Under the int2MR model, the true IV-to-outcome effect in the k-th study is expressed as:

$$\Gamma_{k,j} = (\beta + \beta_{\text{int}} \cdot \rho_k)\, \gamma_j + \alpha_{k,j}, \quad k \in \{0, 1, 2\}, \tag{S6}$$

where γ_j is the true IV-to-exposure effect, and $\alpha_{k,j}$ is the uncorrelated pleiotropic effect specific to study k. The term ρ_k denotes the proportion of comparison-group individuals in the comparison group in the k -th study. In particular, we define $\rho_0 = 0$ for the reference-group-only study, $\rho_1 = 1$ for the comparison-group-only study, and $\rho_2 \in (0, 1)$ for the group-combined study with a known mixed composition.

To demonstrate identifiability, we define a regression slope for each study k as:

$$M_k := \frac{\text{Cov}(\gamma_j, \Gamma_{k,j})}{\text{Var}(\gamma_j)}.$$

Assuming that the IV-exposure effects γ_j are uncorrelated with the pleiotropic effects $\alpha_{k,j}$, i.e., $\mathbb{E}[\gamma_j \alpha_{k,j}] = 0$, it follows that M_k captures the causal slope:

$$M_k = \beta + \beta_{\text{int}} \cdot \rho_k.$$

This relationship yields a system of three linear equations in the two unknowns β and β_{int}:

$$\begin{pmatrix} M_0 \\ M_1 \\ M_2 \end{pmatrix} = \begin{pmatrix} 1 & \rho_0 \\ 1 & \rho_1 \\ 1 & \rho_2 \end{pmatrix} \begin{pmatrix} \beta \\ \beta_{\text{int}} \end{pmatrix}.$$

Provided ρ_0, ρ_1, ρ_2 are distinct by design, the coefficient matrix is of full column rank, yielding a unique solution. Therefore, both β and β_{int} are identifiable from the data. In particular, the causal effect for the reference group is directly given by $M_0 = \beta$, and the interaction effect is obtained as the difference $M_1 - M_0 = \beta_{\text{int}}$.

This identifiability result holds under mild assumptions and underscores a key innovation of int2MR: the ability to disentangle group-specific causal effects using only GWAS summary statistics, provided that the group compositions vary across studies. Throughout this paper, we consider the scenario where two group-specific and one group-combined GWAS datasets are jointly analyzed. To offer more flexibility, heterogeneous ρ_k's are allowed in our software implementation.

S2 Comparison with existing methods

We evaluated our proposed method by comparing it against both individual data-based and summary statistics-based approaches. Since summary statistics-based MR methods cannot accommodate interaction effects, we employed individual data-based methods to assess interaction effects and used summary statistics-based methods to estimate main effects.

Comparison with individual data-based methods. Methods that leverage individual-level data facilitate direct modeling of covariates and their interactions. In our analysis, we compared our approach with two standard methods:

1. Two-Stage Least Squares (2SLS):

In the 2SLS framework [1, 2], the procedure is carried out in two steps. First, the exposure X is predicted from the instrumental variables $\{G_i\}_{i=1}^p$:

$$\text{First-stage regression: } \widehat{X} = \sum_{i=1}^{p} \widehat{\gamma}_i G_i = \mathbb{E}\left(X \mid \{G_i\}_{i=1}^p\right)$$

Next, the outcome Y is regressed on the predicted exposure $\widehat{X}$, the group variable S (e.g., sex or age group), and their interaction:

$$\text{Second-stage regression: } \mathbb{E}\left(Y|\widehat{X}, S\right) = \widehat{\beta} \cdot \widehat{X} + \beta_S \cdot S + \widehat{\beta}_{\text{int}} \cdot \widehat{X} \circ S,$$

This two-step procedure allows for the estimation of both the main effect of X and the interaction effect between X and S.

2. Ordinary Least Squares (OLS) Regression: We also compared our method with a conventional linear regression model that directly incorporates the exposure, the covariate group, and their interaction:

$$\mathbb{E}\left(Y|X, S\right) = \beta \cdot X + \beta_S \cdot S + \beta_{\text{int}} X \circ S.$$

In this OLS approach, all terms (exposure, group, and exposure-group interaction) are included as explanatory variables to capture their joint effect on the outcome Y.

Comparing with summary data-based methods. Although summary-statistic MR methods are computationally efficient and require minimal data sharing, they only estimate overall effects and cannot capture exposure by covariate interactions. Our method overcomes this limitation by enabling the identification and inference of interaction effects using summary data, thereby

extending the capabilities of standard MR approaches that focus solely on total effects.

S3 Supplementary information of simulation

S3.1 Data generation

We simulated summary statistics by generating individual-level genotype and phenotype data for a study setting that included one GWAS of a combined group and two GWASs stratified by subgroup. Let

$$G_X \in \mathbb{R}^{n_X \times p}, \quad G_{Y_k} \in \mathbb{R}^{n_{Y_k} \times p} \quad (k = 0, 1)$$

be the genotype matrices for the exposure GWAS and the two group-specific outcome GWASs, respectively. Let

$$G_{Y_2} \in \mathbb{R}^{n_{Y_2} \times p}$$

denote the genotype matrix for the combined GWAS. Here n_X and n_{Y_k} denote the corresponding sample sizes. Genotype matrices were generated by categorizing continuous genotype data into dosage values $\{0, 1, 2\}$ based on minor allele frequencies (MAF) uniformly distributed in Unif $[0.1, 0.3]$. For each individual in the combined GWAS study, a label vector S was simulated from a Bernoulli distribution Bernoulli(ρ) to indicate the presence of an interaction term, where ρ denotes the probability of being in the group with differential effect. For each SNP $j = 1, \ldots, p$, the effect sizes γ_j were drawn from a uniform distribution on $(-0.2, 0.1) \cup (0.1, 0.2)$, as motivated by previous studies [3].

Individual-level data were generated from the following structural equations:

$$\begin{aligned}
X &= G_X \gamma + U_X + \varepsilon_X, \\
Y_0 &= \beta \cdot X + \alpha_0 \cdot G_{Y_0} + U_{Y_0} + \varepsilon_{Y_0}, \\
Y_1 &= (\beta + \beta_{\text{int}}) \cdot X + \alpha_1 \cdot G_{Y_1} + U_{Y_1} + \varepsilon_{Y_1}, \\
Y_2 &= \beta X + \beta_{\text{int}}(X \circ S) + \alpha_{2,0} \cdot G_{Y_2} \circ (1 - S) + \alpha_{2,1} \cdot G_{Y_2} \circ S + U_{Y_2} + \varepsilon_{Y_2}.
\end{aligned}$$

where X represents the exposure of interest. The vectors $U_X \in \mathbb{R}^{n_X}$ and $U_{Y_k} \in \mathbb{R}^{n_{Y_k}}$ (for $k = 0, 1, 2$) denote the unobserved confounding effects in the IV-to-exposure and IV-to-outcome datasets, respectively. In particular, we assume that the unobserved confounding effect on exposure is represented by U_X, which is generated as a standard normal variable. U_{Y_0} and U_{Y_1} are assumed to consist of independent and identically distributed normal entries with standard deviations U_0 and U_1, respectively. To ensure that the outcome confounders are related to the exposure, we model U_{Y_0} and U_{Y_1} as direct linear functions of U_X. Specifically, we define:

$$U_{Y_0} = U_0 \cdot U_X, , U_{Y_1} = U_1 \cdot U_X, U_{Y_2} = U_0 \cdot U_X \cdot (1 - S) + U_1 \cdot U_X \cdot S.$$

Here, U_0 and U_1 denote the standard deviations for the confounding effects in groups 0 and 1, respectively. This formulation guarantees that the confounders U_{Y_0}, U_{Y_1} and U_{Y_2} are correlated with X via U_X. The terms ε_X and ε_{Y_k} represent random error vectors. The parameter β corresponds to the reference group-specific causal effect, while β_{int} reflects the interaction effect. The operator "$\circ$" indicates element-wise multiplication. The parameters a_0, a_1, $a_{2,0}$, and $a_{2,1}$ modulate the magnitude of the uncorrelated pleiotropy (UHP) effects. In this study, we assume that the pleiotropic effects α_k for the combined GWAS samples in group k are dense and follow an independent standard normal distribution. Moreover, we impose the distributional equivalence

$$a_{2,0} \overset{d}{=} a_0 \quad \text{and} \quad a_{2,1} \overset{d}{=} a_1,$$

so that the UHP effect in the combined GWAS sample represents a mixture of the group-specific effects while remaining independent of the UHP effects in the other GWAS samples.

Finally, we performed a single-variance analysis to obtain summary statistics $\left\{\widehat{\gamma}_j, \widehat{s}_{\gamma_j}\right\}$ for the exposure and $\left\{\widehat{\Gamma}_{k,j}, \widehat{s}_{\Gamma_{k,j}}\right\}$ for the outcome for each SNP $j = 1, \ldots, p$ and k-th GWAS study ($k = 0, 1, 2$). In our simulation setting, we set the sample size of IV-exposure GWAS n_X to be 20000, the sample size of Group 0 n_{Y_0} to be 2000, the sample size of Group 1 n_{Y_1} to be 1000, corresponding to the scenario that sample size from the group of interest is smaller than the reference group. The sample size of the combined group n_{Y_2} may vary.

In the power simulation, we consider two different settings for the main effect β and the interaction effect β_{int}. In the power simulation for β_{int}, we set the true main causal effect $\beta = 0.2$ and the true interaction effect $\beta_{\text{int}} = -0.1$. In simulating the power for the total effect, we set the true main causal effect $\beta = 0.05$ and the true interaction effect $\beta_{\text{int}} = 0$. In both scenarios, we assume that $U_0 = 0.2, U_1 = 0.4$ and that the standard deviation of random noise ε is 1.5. In the power simulation, we assume that there is no uncorrelated pleiotropy. The variance of IVs together explains 37% of the variance of exposure on average in the power simulation. These summary statistics serve as input for our proposed method.

S3.2 Additional simulation results on estimation error

Table A in S1 Text summarizes the root mean squared error (RMSE) to estimate the interaction effect β_{int} and the main effect β in three scenarios: (i) no uncorrelated pleiotropy with mild confounding; (ii) group-specific uncorrelated pleiotropy with mild confounding; and (iii) both uncorrelated pleiotropy and heterogeneous confounding across groups. We compare the basic int2MR method (using only group-specific GWASs), the extended int2MR$_{+20k}$ approach (with a combined sample of 20 000) with competing methods. Simulations follow the type I error rate settings described above, with true effect sizes set to $\beta = 0.1$ and true $\beta_{\text{int}} = -0.05$.

As demonstrated in the main text, int2MR has superior control of the type I error rate. While OLS

and IVW achieve the lowest RMSE for both effects, which reflect their unrestricted fitting that ignores uncorrelated pleiotropy, the int2MR variants impose a variance prior on pleiotropic effects as a robustness regularization penalty. This regularization incurs a marginal RMSE increase but markedly improves standard error calibration. Consequently, both int2MR and int2MR$_{+20k}$ maintain competitive power across all settings while accurately controlling false positives, whereas OLS and IVW systematically underestimate uncertainty under uncorrelated pleiotropy and confounding, leading to inflated type I error rates.

Table A: **Simulation results comparing RMSE of different methods in the presence of horizontal pleiotropy (α) and confounding (U).**

	**RMSE of Interaction Effect β_{int}**					
**Method**	$\alpha = 0$, $U_0 = 0$, $U_1 = 0.2$	$\alpha = 0.02$, $U_0 = 0$, $U_1 = 0.2$	$\alpha = 0.02$, $U_0 = 0.1$, $U_1 = -0.2$	$\alpha_0 = 0$, $\alpha_1 = 0$, $U = 0.2$	$\alpha_0 = 0$, $\alpha_1 = 0.02$, $U = 0.2$	$\alpha_0 = 0.02$, $\alpha_1 = 0.02$, $U = 0.2$
int2MR$_{+20k}$	0.485	0.483	0.469	0.493	0.485	0.484
int2MR	0.504	0.493	0.471	0.503	0.486	0.496
2SLS	0.492	0.484	0.461	0.500	0.497	0.492
OLS	0.512	0.499	0.408	0.492	0.490	0.510
	**RMSE of Main Effect β**					
int2MR$_{+20k}$	0.099	0.098	0.099	0.098	0.099	0.097
int2MR	0.098	0.097	0.098	0.099	0.099	0.102
IVW [4]	0.046	0.065	0.067	0.061	0.063	0.065
MR-Egger [5]	0.454	0.450	0.465	0.450	0.458	0.456
MR-Median [6]	0.399	0.431	0.427	0.399	0.427	0.397
MR-RAPS [7]	0.098	0.100	0.100	0.099	0.100	0.102
MR-cML [3]	0.320	0.352	0.369	0.333	0.341	0.348

S3.3 Additional simulation results on sensitivity to inverse–gamma hyperparameters

We assessed the sensitivity of the Bayesian int2MR method to three near noninformative inverse–gamma priors,

$$\tau^2 \sim \text{IG}(a, b), \quad (a, b) \in \{(0.05, 0.05), (0.02, 0.02), (0.01, 0.01)\},$$

on the empirical type I error rate of the Bayesian int2MR test for $H_0 : \beta_{\text{int}} = 0$. Simulations were conducted under the same three scenarios described above.

Table B in S1 Text displays the type I error rates from these simulations, alongside those from 2SLS and OLS, which do not depend on the specifications of the prior distribution. Across all scenarios,

Bayesian int2MR maintained type I error rates closer to the nominal 5% than competing methods, even when using diffuse priors.

Table B: **Empirical type I error rates (nominal 5%) for Bayesian int2MR, 2SLS and OLS under three inverse–gamma priors.** Top panel (a) reports error rates for testing $H_0 : \beta_{\text{int}} = 0$ (interaction effect); bottom panel (b) reports error rates for testing $H_0 : \beta = 0$ (main effect).

	**Testing $H_0 : \beta_{\text{int}} = 0$**					
**Settings**	$\alpha = 0$, $U_0 = 0$, $U_1 = 0.2$	$\alpha = 0.02$, $U_0 = 0$, $U_1 = 0.2$	$\alpha = 0.02$, $U_0 = 0.1$, $U_1 = -0.2$	$\alpha_0 = 0$, $\alpha_1 = 0$, $U = 0.2$	$\alpha_0 = 0$, $\alpha_1 = 0.02$, $U = 0.2$	$\alpha_0 = 0.02$, $\alpha_1 = 0.02$, $U = 0.2$
IG(0.05, 0.05)	0.034	0.034	0.048	0.036	0.040	0.050
IG(0.02, 0.02)	0.048	0.062	0.066	0.034	0.066	0.070
IG(0.01, 0.01)	0.032	0.076	0.064	0.040	0.042	0.076
2SLS	0.042	0.104	0.080	0.058	0.056	0.106
OLS	0.106	0.138	0.074	0.102	0.106	0.142
	**Testing $H_0 : \beta = 0$**					
IG(0.05, 0.05)	0.024	0.038	0.036	0.028	0.024	0.032
IG(0.02, 0.02)	0.048	0.060	0.066	0.042	0.040	0.068
IG(0.01, 0.01)	0.034	0.060	0.068	0.060	0.034	0.076
2SLS	0.044	0.074	0.074	0.048	0.040	0.088
OLS	0.056	0.062	0.062	0.230	0.254	0.246

S4 Supplementary information of real data applications

S4.1 Additional results on data analysis: identifying risk factors with sex-biased effects on ADHD

To evaluate the robustness of the identified sex-interaction effects on ADHD, we conducted a sensitivity analysis by repeating the int2MR analysis using a more stringent LD clumping threshold ($r^2 = 0.01$) while maintaining the IV significance threshold at 5×10^{-8} as specified in the main analysis. We continued to include the 35 exposures with at least 20 IVs and at most 1000 IVs after IV selection under the original LD clumping threshold ($r^2 = 0.05$), acknowledging that the stricter LD threshold ($r^2 = 0.01$) could reduce the number of IVs for certain exposures. Table C in S1 Text presents the point estimates, p-values, and FDR-adjusted p-values (FDR $\leq$ 10%) for the 15 risk exposures initially identified under $r^2 = 0.05$. Among these 15 exposures, 11 continue to exhibit significant sex-biased interactions with ADHD (p-values < 0.05), indicating that the findings are robust to reasonable variations in LD clumping thresholds.

S4.2 Additional results on data analysis: identifying age-group-specific risk factors for Alzheimer's disease in the oldest-old

This section provides extended analyses that complement the main text by offering a more detailed view of the age-group-specific effects observed in our study. Using data from the Religious Orders Study and Rush Memory and Aging Project (ROSMAP), we applied int2MR to identify risk exposures with differential effects on Alzheimer's disease (AD) pathologies and non-AD pathologies in the oldest-old (age 95+) compared to younger individuals.

In the main text, we highlight that several immune-related traits exhibit significant interaction effects with age on AD pathologies. The analyses presented here further illustrate these group-specific differences by showing separate heatmaps for non-AD and AD pathologies in the oldest-old group (95+) and younger group (95−).

To verify that our age-group-specific interaction effects on AD are not driven by the choice of LD clumping threshold, we performed a sensitivity analysis by rerunning int2MR in a more stringent clumping setting ($r^2 = 0.01$), while keeping the genome-wide significance threshold at 5×10^{-8}. Fig B in S1 Text compares the heatmaps of effect estimates on non-AD (top panels) and AD pathologies (bottom panels) between the primary analysis ($r^2 = 0.05$) and the sensitivity analysis ($r^2 = 0.01$). The overall pattern of age-dependent interaction effects is preserved, confirming the robustness of our findings with respect to LD clumping parameters.

To further demonstrate the consistency of our findings, we summarize the numerical results of the primary and sensitivity analyses in Table D in S1 Text. This table reports the estimated interaction effects of top exposures across three AD pathologies under both LD clumping thresholds ($r^2 = 0.05$ and $r^2 = 0.01$). Despite a reduction in the number of instruments under the stricter threshold, the direction and magnitude of the estimated interaction effects remain largely stable for most traits. Notably, key immune-related exposures maintain statistical significance in both settings, supporting the robustness of our age-dependent findings.

In addition to the pathology-based analyses, we also applied the int2MR method to investigate age-dependent interaction effects for clinically diagnosed Alzheimer's disease (AD). Table E in S1 Text summarizes the estimated interaction effects ($\widehat{\beta}_{\text{int}}$), along with their p-values and FDR-adjusted p-values, for the top-ranked exposures identified in this analysis with FDR-adjusted p-values < 0.05. Notably, traits such as fluid intelligence score, atrial fibrillation, and several immune- and inflammation-related traits show significant interaction effects with age in influencing the risk of clinical AD. These findings reinforce the consistency of our conclusions across both pathological and clinical definitions of AD.

We applied two conventional Mendelian randomization methods, IVW and MR-Egger, to the full-population GWAS summary statistics in order to estimate the overall exposure effects on Alzheimer's disease. The resulting Z-scores are displayed in Fig C in S1 Text. When compared with

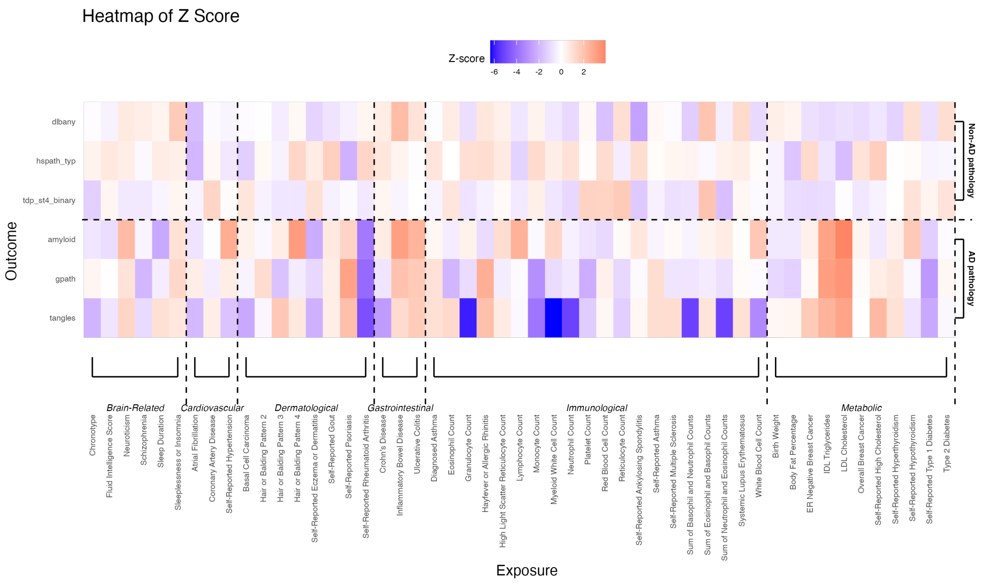

(a)

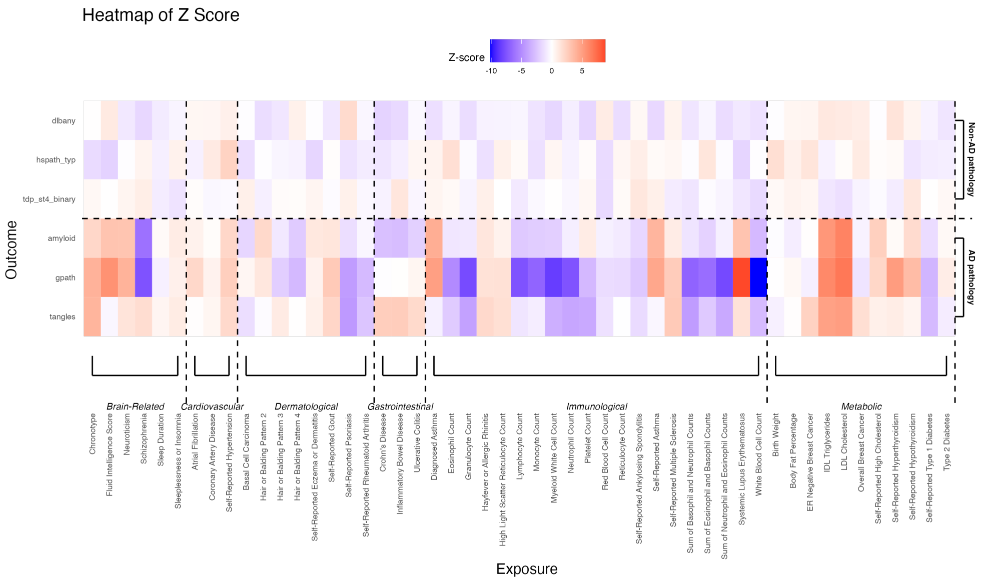

(b)

Fig A: **Two heatmaps showing the Z-scores of the age-group specific effects on AD pathologies (bottom panel) and non-AD pathologies in the oldest-old group versus the younger group.** Here, (a) shows the age-group specific effects in the oldest-old group (death-age 95+), and (b) shows the age-group specific effects in the younger age group (death-age 95-).

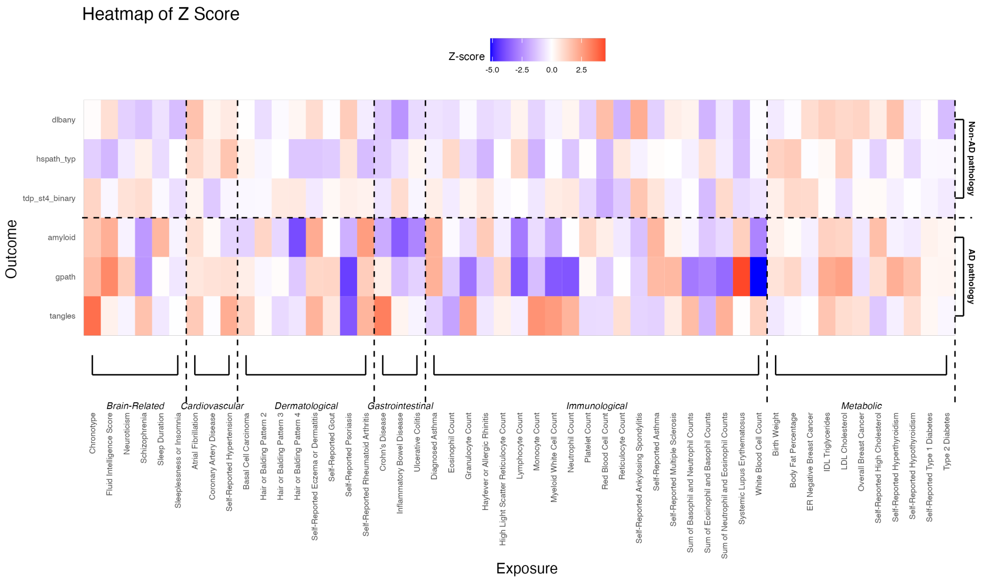

(a)

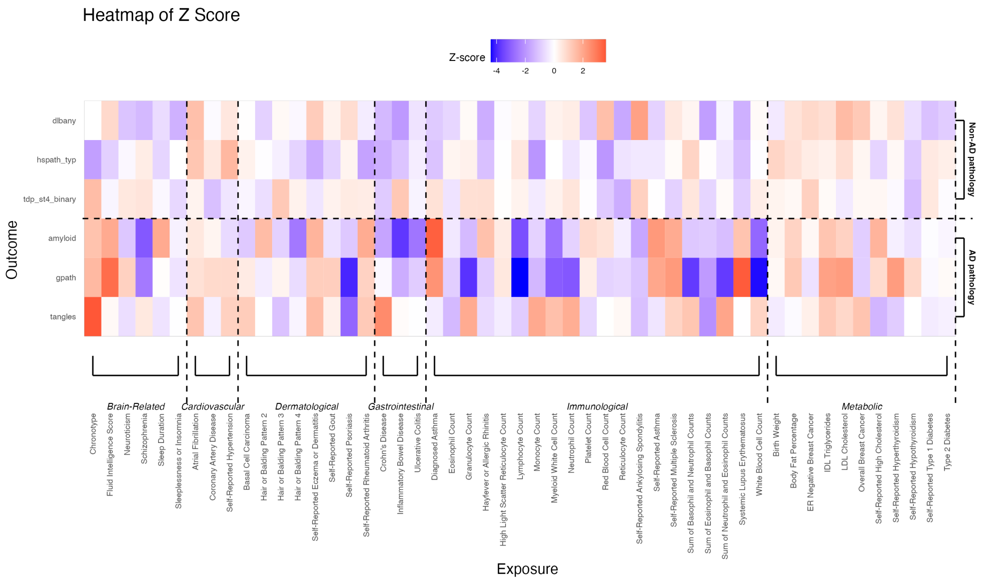

(b)

Fig B: **Two heatmaps that compare the interaction effect estimates between the primary analysis ($r^2 = 0.05$, the upper one) and the sensitivity analysis ($r^2 = 0.01$, the lower one).** Note: (a) here corresponds to Fig 4 in the main text. The distributions of Z-scores across exposures remain largely unchanged.

the int2MR-derived Z-scores (Fig A in S1 Text), we observe a strong concordance, particularly with the younger (95-) subgroup (Fig Ab in S1 Text). This alignment likely reflects the fact that the oldest-old group represents only a small fraction (408/2587) of the full sample, causing traditional MR estimates in the overall cohort to be driven predominantly by the larger, younger majority.

Similarly, we applied IVW and MR–Egger solely to the oldest-old GWAS summary statistics to estimate exposure effects on Alzheimer's disease within this subgroup. The resulting Z-scores are presented in Fig D in S1 Text. When these traditional MR heatmaps are compared with those obtained from our int2MR method, which integrates GWAS summary statistics from both the oldest-old group and the full population (Fig Aa in S1 Text), the Z-score distributions across exposures remain largely consistent.

S5 Algorithmic details

This section outlines the Bayesian hierarchical model implemented in the int2MR method and describes the No-U-Turn Sampler (NUTS) used for efficient parameter estimation and inference [8]. We begin by introducing a generalized MR model, which accommodates an arbitrary number of GWAS datasets and allows for flexible ρ.

S5.1 Bayesian hierarchical model with independent SNPs

The proposed method generalizes to scenarios involving K GWAS datasets for instrument-to-outcome associations. Suppose we have summary statistics from K IV-to-outcome GWASs and one exposure GWAS. For the j-th SNP, the model is expressed as:

$$\begin{pmatrix} \widehat{\Gamma}_{1,j} \\ \widehat{\Gamma}_{2,j} \\ \vdots \\ \widehat{\Gamma}_{K,j} \\ \widehat{\gamma}_j \end{pmatrix} \sim \mathcal{N}\left(\begin{pmatrix} \Gamma_{1,j} \\ \Gamma_{2,j} \\ \vdots \\ \Gamma_{K,j} \\ \gamma_j \end{pmatrix}, \operatorname{diag}\left(\widehat{s}^2_{1,j}, \ldots, \widehat{s}^2_{K,j}, \widehat{s}^2_j\right) \right),$$

where $j = 1, 2, \ldots, p$. The latent effects $\Gamma_{k,j}$, $\Gamma_{0,j}$, and γ_j satisfy the structural equation:

$$\Gamma_{k,j} = (\beta + \rho_k \cdot \beta_{\text{int}}) \cdot \gamma_j + \alpha_{k,j}, \quad k = 1, 2, \ldots, K.$$

Here, γ_j represents the true instrument-exposure effect, while $\Gamma_{k,j}$ represents the true instrument-outcome effects for group-specific GWAS datasets. To capture uncorrelated pleiotropy (UHP), the

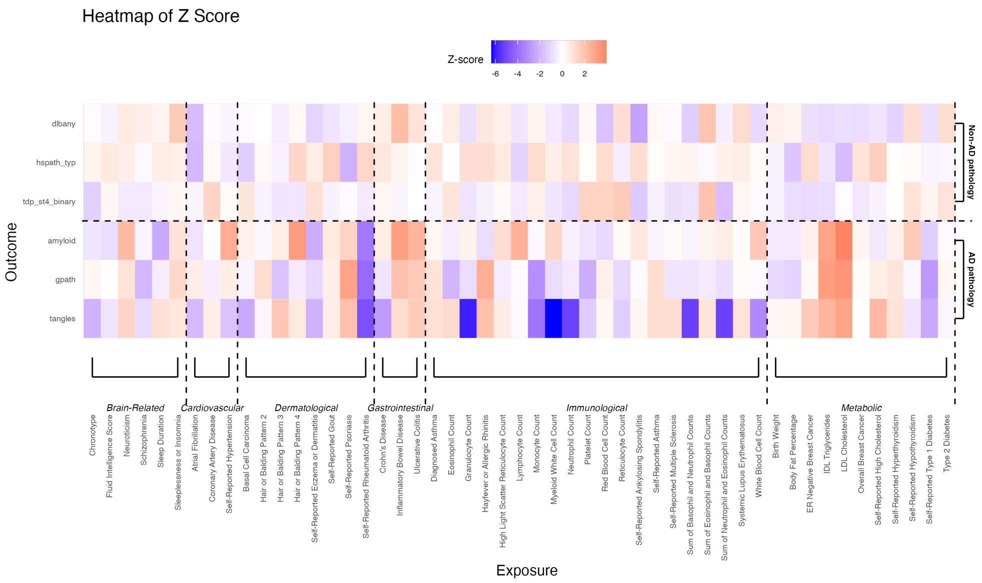

(a)

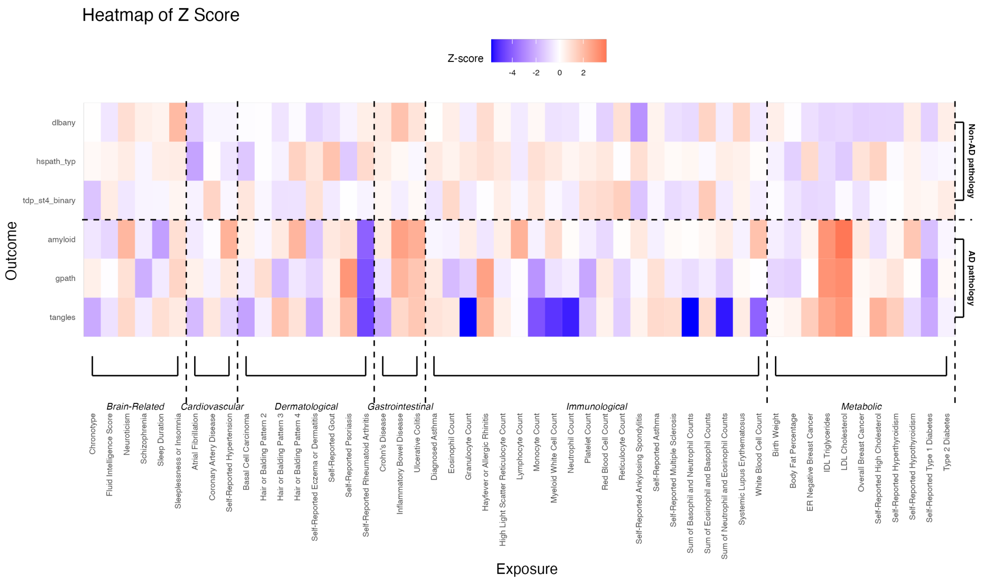

(b)

Fig C: **Two heatmaps showing the Z-scores of the total effects on AD pathologies (bottom panel) and non-AD pathologies (top panel) based solely on the GWAS summary statistics for the full population.** Here, (a) shows the Z-scores estimated by IVW, and (b) shows the Z-scores estimated by MR-Egger.

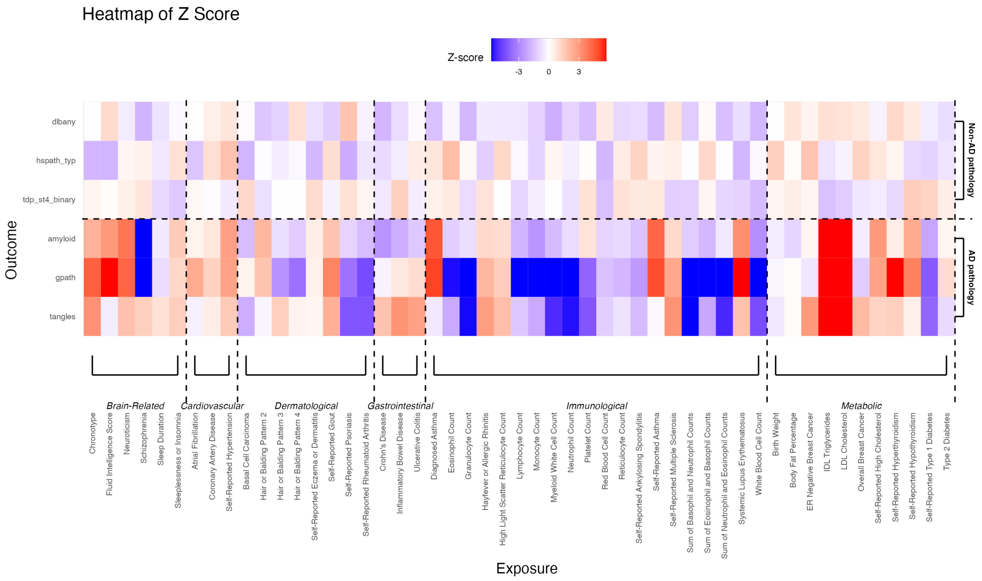

(a)

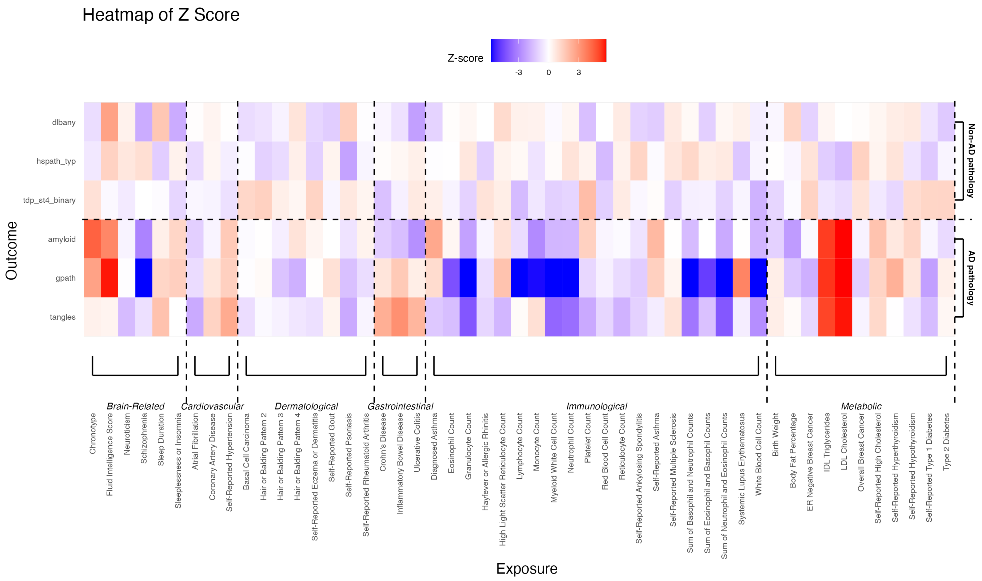

(b)

Fig D: **Two heatmaps showing the Z-scores of the oldest-old group-specific effects on AD pathologies (bottom panel) and non-AD pathologies (top panel) based solely on the GWAS summary statistics for the oldest-old group.** Here, (a) shows the Z-scores estimated by IVW, and (b) shows the Z-scores estimated by MR-Egger.

SNP-specific pleiotropic effects, $\alpha_{k,j}$, are modeled as:

$$\alpha_{k,j} \sim \mathcal{N}(0, \sigma^2_{\alpha,k}).$$

S5.2 Posterior likelihood specification

The full-data posterior likelihood is denoted as:

$$L\left(\Theta \mid \widehat{\gamma}, \widehat{s}_\gamma, \widehat{\Gamma}_k, \widehat{s}_{\Gamma_k}\right),$$

where the parameters are:

$$\Theta = \left(\beta, \beta_{\text{int}}, \{\gamma_j\}_{j=1}^p, \{\alpha_{k,j}\}_{k=1,\dots,K; j=1,\dots,p}\right).$$

Assuming independence across SNPs, the likelihood decomposes as:

$$L(\Theta) \propto \prod_{k=1}^{K} \prod_{j=1}^{p} \mathbf{p}\left(\widehat{\Gamma}_{k,j} \mid \Gamma_{k,j}, \widehat{s}_{\Gamma_{k,j}}\right) \cdot \mathbf{p}\left(\widehat{\gamma}_j \mid \gamma_j, \widehat{s}_{\gamma_j}\right) \cdot \mathbf{p}\left(\Gamma_{k,j} \mid \gamma_j, \alpha_{k,j}; \beta, \beta_{\text{int}}\right) \cdot \pi(\Theta).$$

Here, $\pi(\Theta)$ incorporates the priors on all parameters and hyperparameters, including inverse-Gamma priors for variance terms and normal priors for latent effects. For instance:

$$\sigma^2_{\alpha,k} \sim \text{IG}(\alpha_{\alpha,k}, \beta_{\alpha,k}).$$

S5.3 Choice of priors and hyperparameters

The variance parameters $\sigma^2_{\alpha,k}$ are modeled using inverse-Gamma priors:

$$\sigma^2_{\alpha,k} \sim \text{IG}(\alpha_{\alpha,k}, \beta_{\alpha,k}),$$

with hyperparameters $\alpha_{\alpha,k} = \beta_{\alpha,k} = 2 \times 10^{-2}$ to ensure non-informative priors, as recommended by Gelman [9]. Equivalently, in a Maximum a posteriori (MAP) formulation, this prior contributes to the penalty

$$(\alpha_{\alpha,k} + 1) \log \sigma^2_{\alpha,k} + \frac{\beta_{\alpha,k}}{\sigma^2_{\alpha,k}}$$

which discourages both overly large and overly small estimates of $\sigma^2_{\alpha,k}$.

For the latent effect γ_j, we assume that $\gamma_j \sim \mathcal{N}(0, \sigma^2_\gamma)$. Here, the prior variance σ^2_γ is the variance

of $\hat{\gamma}$ across all SNPs. Furthermore, we assume that

$$\sigma_\gamma^2 \sim \mathrm{IG}(\alpha_\gamma, \beta_\gamma)$$

with hyperparameters $\alpha_\gamma = \beta_\gamma = 2 \times 10^{-2}$.

Furthermore, we assign uniform priors to the causal effect parameters

$$\pi(\beta), \pi(\beta_{\text{int}}) \propto 1.$$

This choice represents a non-informative approach, ensuring that the inference of parameters of interest is driven solely by the observed data.

S5.4 Posterior distribution and benefit of data integration

Let $\Theta = \left(\beta, \beta_{\text{int}}, \{\gamma_j\}_{j\in[p]}, \{\alpha_{k,j}\}_{k\in\{0,1,2\}}\right)$. Here, we use $\{\widetilde{\sigma}_{\alpha,k}\}_{k\in\{0,1,2\}}$ to denote the true variance of the uncorrelated pleiotropy effects $\{\alpha_{k,j}\}_{k\in\{0,1,2\}}$. The joint likelihood of the observed summary statistics factorizes as

$$L(\Theta) \propto \prod_{k\in\{0,1,2\}} \prod_{j=1}^{p} \mathbf{p}\left(\widehat{\Gamma}_{k,j} \mid \Gamma_{k,j}, \widehat{s}_{\Gamma_{k,j}}\right) \cdot \mathbf{p}\left(\widehat{\gamma}_j \mid \gamma_j, \widehat{s}_{\gamma_j}\right) \cdot \mathbf{p}\left(\Gamma_{k,j} \mid \gamma_j, \alpha_{k,j}; \beta, \beta_{\text{int}}\right) \cdot \pi(\Theta).$$

After substituting the normal error models and the inverse-Gamma priors, one obtains an exponentiated quadratic form in $\{\alpha_{k,j}\}_{k\in\{0,1,2\},j\in[p]}$ and $\{\gamma_j\}_{j\in[p]}$.

$$\begin{aligned} L(\Theta) \propto &\exp\left(-\sum_{j=1}^{p} \frac{1}{2\widehat{s}_{0,j}^2}\left(\widehat{\Gamma}_{0,j} - \beta\cdot\gamma_j - \alpha_{0,j}\right)^2\right) \cdot \exp\left(-\sum_{j=1}^{p} \frac{1}{2\widehat{s}_{1,j}^2}\left(\widehat{\Gamma}_{1,j} - (\beta+\beta_{\text{int}})\cdot\gamma_j - \alpha_{1,j}\right)^2\right) \\ &\cdot \exp\left(-\sum_{j=1}^{p} \frac{1}{2\widehat{s}_{2,j}^2}\left(\widehat{\Gamma}_{2,j} - (\beta+\rho\cdot\beta_{\text{int}})\cdot\gamma_j - \alpha_{2,j}\right)^2\right) \cdot \exp\left(-\sum_{j=1}^{p} \frac{1}{2\widehat{s}_{\gamma,j}^2}\left(\widehat{\gamma}_j - \gamma_j\right)^2\right) \\ &\cdot \exp\left(-\frac{1}{2\widetilde{\sigma}_{\alpha,0}^2}\sum_{j=1}^{p}\alpha_{0,j}^2\right) \cdot \exp\left(-\frac{1}{2\widetilde{\sigma}_{\alpha,1}^2}\sum_{j=1}^{p}\alpha_{1,j}^2\right) \cdot \exp\left(-\frac{1}{2\widetilde{\sigma}_{\alpha,2}^2}\sum_{j=1}^{p}\alpha_{2,j}^2\right) \cdot \exp\left(-\frac{1}{2\widetilde{\sigma}_{\gamma}^2}\sum_{j=1}^{p}\gamma_j^2\right). \end{aligned}$$

Then, our goal is to integrate the latent variables $\alpha_{0,j}$, $\alpha_{1,j}$, $\alpha_{2,j}$ and γ_j. Note that

$$\mathcal{N}\left(\widehat{\Gamma} \mid \mu, s^2 + \bar{\sigma}^2\right) \propto \int \mathcal{N}\left(\widehat{\Gamma} \mid \mu + \alpha, s^2\right) \mathcal{N}\left(\alpha \mid 0, \bar{\sigma}^2\right) d\alpha.$$

It implies that integrating out each $\alpha_{k,j}$ analytically replaces its contribution $\widehat{s}_{k,j}^2$ by the augmented

variance $\widetilde{s}_{k,j}^2 + \widetilde{\sigma}_{\alpha,k}^2$ for any $k = 0, 1, 2$:

$$L(\Theta) \propto \exp\left(-\sum_{j=1}^{p} \frac{1}{2\left(\widehat{s}_{0,j}^2 + \widetilde{\sigma}_{\alpha,0}^2\right)} \left(\widehat{\Gamma}_{0,j} - \beta \cdot \gamma_j\right)^2\right) \cdot \exp\left(-\sum_{j=1}^{p} \frac{1}{2\left(\widehat{s}_{1,j}^2 + \widetilde{\sigma}_{\alpha,1}^2\right)} \left(\widehat{\Gamma}_{1,j} - (\beta + \beta_{\text{int}}) \cdot \gamma_j\right)^2\right)$$
$$\cdot \exp\left(-\sum_{j=1}^{p} \frac{1}{2\left(\widehat{s}_{2,j}^2 + \widetilde{\sigma}_{\alpha,2}^2\right)} \left(\widehat{\Gamma}_{2,j} - (\beta + \rho \cdot \beta_{\text{int}}) \cdot \gamma_j\right)^2\right)$$
$$\cdot \exp\left(-\sum_{j=1}^{p} \frac{1}{2\widehat{s}_{\gamma,j}^2} \left(\widehat{\gamma}_j - \gamma_j\right)^2\right) \cdot \exp\left(-\frac{1}{2\widetilde{\sigma}_{\gamma}^2} \sum_{j=1}^{p} \gamma_j^2\right).$$

Here, $\widetilde{\sigma}_{\alpha,k}^2$ can be viewed as the ground-truth variance component of the uncorrelated pleiotropic effects. In practice, we assumes an inverse-gamma distribution on $\widetilde{\sigma}_{\alpha,k}^2$, which is the conjugate prior for variance component. It follows that the 2×2 precision matrix conditioning on the true γ_j's is

$$I\left(\beta, \beta_{\text{int}} \mid \{\gamma_j\}_{j=1}^{p}\right) = \sum_{j=1}^{p} \gamma_j^2 \begin{pmatrix} \frac{1}{\widetilde{s}_{0,j}^2} + \frac{1}{\widetilde{s}_{1,j}^2} + \frac{1}{\widetilde{s}_{2,j}^2} & \frac{1}{\widetilde{s}_{1,j}^2} + \rho \frac{1}{\widetilde{s}_{2,j}^2} \\ \frac{1}{\widetilde{s}_{1,j}^2} + \rho \frac{1}{\widetilde{s}_{2,j}^2} & \frac{1}{\widetilde{s}_{1,j}^2} + \rho^2 \frac{1}{\widetilde{s}_{2,j}^2} \end{pmatrix},$$

where $\widetilde{s}_{k,j}^2 = \widehat{\sigma}_{k,j}^2 + \widetilde{\sigma}_{\alpha,k}^2$ for any $k = 0, 1, 2$.

In contrast, if we only observe two group-specific GWAS studies, the likelihood would be

$$L_0(\Theta) \propto \exp\left(-\sum_{j=1}^{p} \frac{1}{2\left(\widehat{s}_{0,j}^2 + \widetilde{\sigma}_{\alpha,0}^2\right)} \left(\widehat{\Gamma}_{0,j} - \beta \cdot \gamma_j\right)^2\right) \cdot \exp\left(-\sum_{j=1}^{p} \frac{1}{2\left(\widehat{s}_{1,j}^2 + \widetilde{\sigma}_{\alpha,1}^2\right)} \left(\widehat{\Gamma}_{1,j} - (\beta + \beta_{\text{int}}) \cdot \gamma_j\right)^2\right)$$
$$\cdot \exp\left(-\sum_{j=1}^{p} \frac{1}{2\widehat{s}_{\gamma,j}^2} \left(\widehat{\gamma}_j - \gamma_j\right)^2\right) \cdot \exp\left(-\frac{1}{2\widetilde{\sigma}_{\gamma}^2} \sum_{j=1}^{p} \gamma_j^2\right).$$

Incorporating the combined GWAS contributes an additional information term to the precision matrix:

$$I = I_0 + \sum_{j=1}^{p} \frac{\gamma_j^2}{\widetilde{s}_{2,j}^2} \begin{pmatrix} 1 & \rho \\ \rho & \rho^2 \end{pmatrix} > I_0,$$

where

$$I_0 = \sum_{j=1}^{p} \gamma_j^2 \begin{pmatrix} \frac{1}{\widetilde{s}_{0,j}^2} + \frac{1}{\widetilde{s}_{1,j}^2} & \frac{1}{\widetilde{s}_{1,j}^2} \\ \frac{1}{\widetilde{s}_{1,j}^2} & \frac{1}{\widetilde{s}_{1,j}^2} \end{pmatrix},$$

is the precision based solely on group-separated summary statistics. Intuitively, this augmentation improves the estimation precision of both β and β_{int}. To formalize our arguments, let

$$D^{(2)} = \left\{\widehat{\Gamma}_{0,j}, \widehat{\Gamma}_{1,j}, \widehat{\gamma}_j\right\}_{j=1}^{p}, \quad D^{(3)} = D^{(2)} \cup \left\{\widehat{\Gamma}_{2,j}\right\}_{j=1}^{p},$$

and write $\Sigma^{(2)} = \operatorname{Cov}\big[(\beta, \beta_{\text{int}}) \mid D^{(2)}\big]$ and $\Sigma^{(3)} = \operatorname{Cov}\big[(\beta, \beta_{\text{int}}) \mid D^{(3)}\big]$, where in both posteriors all nuisance γ_j have been integrated out. Then by the law of total covariance (conditioning first on $D^{(2)}$ and then further on the third-study data $\{\widehat{\Gamma}_{2,j}\}$) one has

$$\Sigma^{(2)} = \; E\big[\operatorname{Cov}\big((\beta, \beta_{\text{int}}) \mid D^{(2)}, \{\widehat{\Gamma}_{2,j}\}\big) \;\big|\; D^{(2)}\big] \;+\; \operatorname{Cov}\big(E\big[(\beta, \beta_{\text{int}}) \mid D^{(2)}, \{\widehat{\Gamma}_{2,j}\}\big]\,, \;\big|\; D^{(2)}\big).$$

Since the second term is positive semidefinite, $\Sigma^{(2)} \succeq E[\operatorname{Cov}((\beta, \beta_{\text{int}}) \mid D^{(3)}) \mid D^{(2)}] = \Sigma^{(3)}$, i.e.

$$\operatorname{Cov}\big[(\beta, \beta_{\text{int}}) \mid D^{(3)}\big] \;\preceq\; \operatorname{Cov}\big[(\beta, \beta_{\text{int}}) \mid D^{(2)}\big].$$

Hence, integrating out the $\{\gamma_j\}_{j \in [p]}$ and comparing the two- and three-study posteriors shows that adding the third GWAS strictly reduces the posterior covariance of $(\beta, \beta_{\text{int}})$.

S5.5 Implementation details

Efficient posterior sampling is critical in high-dimensional MR analyses. Traditional MCMC methods like Gibbs sampling [10] often face challenges such as slow convergence and poor mixing. To overcome these limitations, we use the No-U-Turn Sampler (NUTS), an adaptive extension of Hamiltonian Monte Carlo (HMC). NUTS dynamically tunes the trajectory length, reducing the need for manual tuning and enhancing computational efficiency in high-dimensional parameter spaces. We refer readers to The No-U-Turn Sampler paper for technical details [8].

We ran two NUTS chains, each with 5,000 warm-up and 5,000 sampling iterations. We replicated each simulation 1,000 times to evaluate type I error rates and power, as reported in the main and supplementary results. To speed up computation in intensive scenarios, an optimization-based option to perform maximum a posteriori (MAP) estimation is also provided and recommended for computationally intensive scenarios. The optimization-based method directly finds the maximum a posteriori (MAP) estimation, i.e., posterior mode, by maximizing the joint posterior likelihood while the sampling based method uses the posterior mean as the point estimates. By Bernstein–von Mises theorem, the posterior distribution converges to normal distribution asymptotically. Therefore, MAP estimation and sampling-based method, which finds posterior mean, are asymptotically equivalent. In this study, we focus exclusively on the sampling-based approach; validation of the MAP alternative is left for future work.

References

[1] Basmann RL. A generalized classical method of linear estimation of coefficients in a structural equation. Econometrica: Journal of the Econometric Society. 1957; p. 77–83.

[2] Zhu X, Yang Y, Lorincz-Comi N, Li G, Bentley AR, de Vries PS, et al. An approach to identify gene-environment interactions and reveal new biological insight in complex traits. Nature Communications. 2024;15(1):3385.

[3] Xue H, Shen X, Pan W. Constrained maximum likelihood-based Mendelian randomization robust to both correlated and uncorrelated pleiotropic effects. Am J Hum Genet. 2021;108(7):1251–1269.

[4] Burgess S, Butterworth A, Thompson SG. Mendelian randomization analysis with multiple genetic variants using summarized data. Genet Epidemiol. 2013;37(7):658–665.

[5] Bowden J, Davey Smith G, Burgess S. Mendelian randomization with invalid instruments: effect estimation and bias detection through Egger regression. Int J Epidemiol. 2015;44(2):512–525.

[6] Bowden J, Davey Smith G, Haycock PC, Burgess S. Consistent estimation in Mendelian randomization with some invalid instruments using a weighted median estimator. Genetic epidemiology. 2016;40(4):304–314.

[7] Zhao Q, Wang J, Hemani G, Bowden J, Small DS, et al. Statistical inference in two-sample summary-data Mendelian randomization using robust adjusted profile score. Annals of Statistics. 2020;48(3):1742–1769.

[8] Hoffman MD, Gelman A, et al. The No-U-Turn sampler: adaptively setting path lengths in Hamiltonian Monte Carlo. J Mach Learn Res. 2014;15(1):1593–1623.

[9] Gelman A. Prior distributions for variance parameters in hierarchical models (comment on article by Browne and Draper). Bayesian Analysis. 2006;1(3):515 – 534.

[10] Grant AJ, Burgess S. A Bayesian approach to Mendelian randomization using summary statistics in the univariable and multivariable settings with correlated pleiotropy. The American Journal of Human Genetics. 2024;111(1):165–180.

Table C: **Sensitivity Analysis of ADHD Sex-Interaction Estimates to LD Clumping Threshold ($r^2 = 0.01$).** Eleven exposures with FDR $\leq$ 10% under $r^2 = 0.05$ remain significant (p-values < 0.05) under $r^2 = 0.01$.

**Exposure**	$r^2 = 0.01$					$r^2 = 0.05$				
	#IVs	**+ comb.**		**strat.**		#IVs	**+ comb.**		**strat.**	
		$\widehat{\beta}_{\text{int}}$	p-value	$\widehat{\beta}_{\text{int}}$	p-value		$\widehat{\beta}_{\text{int}}$	p-value	$\widehat{\beta}_{\text{int}}$	p-value
High Light Scatter Reticulocyte Count	536	0.094	0.0222	0.084	0.0489	676	0.125	0.0013	0.117	0.0021
Reticulocyte count	539	0.085	0.0358	0.075	0.0529	681	0.100	0.0036	0.089	0.0147
Sum of Neutrophil and Eosinophil Counts	372	0.134	0.0033	0.112	0.0216	412	0.124	0.0044	0.113	0.0146
White Blood Cell Count	451	0.109	0.0046	0.101	0.0104	503	0.110	0.0037	0.101	0.0042
Hayfever or Allergic Rhinitis	303	-0.426	0.0073	-0.366	0.0221	353	-0.412	0.0045	-0.371	0.0095
Self-Reported Hypertension	394	-0.432	0.0083	-0.424	0.0069	407	-0.415	0.0065	-0.412	0.0086
Self-Reported Psoriasis	151	0.219	0.0076	0.226	0.0060	190	0.221	0.0072	0.210	0.0105
Granulocyte Count	369	0.115	0.0137	0.107	0.0268	409	0.125	0.0110	0.117	0.0100
Myeloid White Cell Count	376	0.125	0.0062	0.114	0.0199	418	0.102	0.0143	0.096	0.0257
Eosinophil Count	495	0.057	0.1741	0.055	0.1490	601	0.080	0.0198	0.078	0.0296
Neutrophil Count	369	0.114	0.0161	0.104	0.0396	418	0.117	0.0198	0.104	0.0343
Basophil Neutrophil Count	373	0.108	0.0366	0.100	0.0416	415	0.112	0.0231	0.108	0.0302
Lymphocyte Count	480	0.045	0.2911	0.041	0.3195	556	0.082	0.0275	0.078	0.0375
Sum of Eosinophil and Basophil Counts	464	0.028	0.4721	0.028	0.4217	560	0.075	0.0601	-0.080	0.0333
Inflammatory Bowel Disease	142	-0.020	0.3107	-0.024	0.2656	155	-0.027	0.1997	-0.039	0.0337

Note. "+ comb." = + sex-combined GWAS; "strat." = sex-stratified GWAS only.

Table D: **Sensitivity analysis of int2MR interaction effect estimates across LD clumping thresholds ($r^2 = 0.05$ vs. $r^2 = 0.01$).** Comparison of $\widehat{\beta}_{\text{int}}$ estimates and p-values across exposures for three AD pathologies. The results show that the direction and magnitude of age-group interaction effects are largely consistent across thresholds.

	$r^2 = 0.05$			$r^2 = 0.01$		
**Exposure**	#IVs	$\widehat{\beta}_{\text{int}}$	p-value	#IVs	$\widehat{\beta}_{\text{int}}$	p-value
**Amyloid Pathology**						
Hair or Balding Pattern 4	710	-0.5482	6.20×10^{-5}	604	-0.4967	1.02×10^{-2}
Inflammatory Bowel Disease	243	-0.2078	2.70×10^{-4}	232	-0.1947	1.70×10^{-4}
**Global AD Pathology Burden**						
White Blood Cell Count	833	-0.2355	2.35×10^{-7}	749	-0.2365	1.60×10^{-5}
Systemic Lupus Erythematosus	251	0.0630	6.80×10^{-6}	215	0.0553	4.50×10^{-4}
Myeloid White Cell Count	686	-0.2135	1.80×10^{-4}	605	-0.2288	1.90×10^{-3}
Neutrophil Count	660	-0.2128	1.40×10^{-4}	584	-0.2378	1.43×10^{-3}
Sum of Neutrophil & Eosinophil Counts	656	-0.2056	8.25×10^{-3}	582	-0.2191	2.06×10^{-4}
Granulocyte Count	651	-0.2110	1.82×10^{-3}	575	-0.2184	1.18×10^{-4}
Fluid Intelligence	174	0.2522	1.83×10^{-3}	174	0.2514	1.41×10^{-3}
Sum of Basophil & Neutrophil Counts	659	-0.2138	2.83×10^{-3}	587	-0.2379	2.06×10^{-4}
Sum of Eosinophil & Basophil Counts	934	-0.1353	5.98×10^{-2}	781	-0.1115	5.70×10^{-2}
**Tangles Pathology**						
Chronotype	292	1.3546	2.00×10^{-4}	288	1.6388	3.40×10^{-4}
Self-Reported Psoriasis	315	-0.6228	1.9×10^{-5}	259	-1.219	3.55×10^{-3}
Crohn's Disease	192	0.1629	8.00×10^{-4}	177	0.1523	9.57×10^{-3}

Table E: **Risk exposures and their age-group interaction effect estimates obtained by int2MR for clinically diagnosed AD.** Significant exposure-by-age-group interactions for clinically diagnosed Alzheimer's disease (AD) identified using the int2MR method. Displayed are the interaction effect estimates $\widehat{\beta}_{\text{int}}$, p-values, and FDR-adjusted p-values.

**Exposure**	**#IVs**	$\widehat{\beta}_{\textbf{int}}$	p**-value**	**FDR adj.**
Fluid Intelligence Score	172	1.5948	4.76×10^{-8}	1.62×10^{-6}
Systemic Lupus Erythematosus	250	0.2151	1.14×10^{-7}	2.57×10^{-6}
Self-Reported Hypertension	683	2.8538	6.01×10^{-7}	1.02×10^{-5}
Neuroticism	265	1.0031	1.95×10^{-6}	2.65×10^{-5}
Inflammatory Bowel Disease	242	0.2573	0.00080	0.00904
Crohn's Disease	190	0.2560	0.00152	0.01322
Sum of Eosinophil and Basophil Counts	925	−0.5003	0.00156	0.01322
Hair or Balding Pattern 4	708	−1.1274	0.00188	0.01323
Self-Reported Eczema or Dermatitis	80	6.9056	0.00214	0.01323
Self-Reported Psoriasis	313	−1.4041	0.00382	0.02111
Self-Reported Hypothyroidism	528	2.0568	0.00441	0.02111
Hayfever or Allergic Rhinitis	543	−2.0306	0.00452	0.02111
Atrial Fibrillation	476	0.4380	0.00466	0.02111
