# Supplementary material for: Integrative Mendelian randomization for detecting exposure-by-group interactions using group-specific and combined summary statistics
Source: PLoS Genet. 2025 Sep 11;21(9):e1011819. doi: 10.1371/journal.pgen.1011819 (PMC12440225; doi:10.1371/journal.pgen.1011819)
Supplement: Attachment — Submitted filename: response_letter.pdf [file pgen.1011819.s005.pdf]

From: Lin S. Chen

Department of Public Health Sciences

The University of Chicago

Chicago, Illinois 60637

May 5th, 2025

Dear Editorial Board of *PLoS Genetics*,

We are writing to submit the revised manuscript entitled “Integrative Mendelian randomization for detecting exposure-by-group interactions using group-specific and combined summary statistics”.

We would like to thank the editor and three reviewers for their thorough and constructive comments and suggestions. We have carefully addressed each reviewer’s comments in a comprehensive, point-by-point manner, as outlined in the accompanying document. The reviewer’s comments are presented in italic, followed by our detailed responses. We have revised the manuscript and Supplementary Material S1 Appendix accordingly. We believe the manuscript is substantially improved after revision.

Please note that Figure 1 (Illustration) is drafted using Microsoft Powerpoint icons. According to “https://support.microsoft.com/en-us/office/insert-icons-in-microsoft-365-e2459f17-3996-4795-996e-b9a13486fa79", “these icons are free to use and there’s no royalty or copyright to worry about."

Thank you for your consideration, and please feel free to contact us if you need any further information.

Sincerely yours,

Lin S. Chen and co-authors

Point-by-Point Response for Reviewer 1

Comment 1: *It might be better to move the model detail in the Supplementary S1.1 to the main text. This is insightful to motivate the main relationship of GWAS effects. There are several typos in the derivation, e.g. the equation after S2, p should be ρ, and RHS is not conditioning on X.*

Response to Comment 1:

We have moved the models in Supplementary S1.1 to the main text. Specifically in the main text, we added a new subsection titled "Modeling interaction effects in the group-combined GWAS: from individual-level data to GWAS summary statistics" in the section "Description of Methods" (see Lines 53-80). This subsection explains the interaction effect in the group-combined GWAS. It connects with the previous subsection introducing the MR model using group-separated GWAS to identify interaction effects, and the following subsection presenting our proposed MR model using both group-separated and group-combined GWAS summary statistics.

Additionally, we have corrected the typos. The symbol p has been replaced with ρ, and the right-hand side of the equation is now presented without conditioning on X.

Comment 2: *Related to 1, could the authors provide more details on the assumptions and identifiability conditions of the model?*

a) For example, what is the definition of valid/invalid IVs? What are the conditions such that β and β_{int} are identifiable? The current model implicitly assume the group specific genetic effect on the outcome only due to the group specific effect of the exposure on the outcome (if no pleiotropy is present), is this correct? Group specific genetic effect on the exposure is not allowed?

Response to Comment 2a):

In the Section "Model Idenitifiability" of the Supplementary Material (see Page 4, Lines 5-29), we add a precise proof of the identifiability condition of the main effect β and the interaction effect β_{int} and thereby conclude that

"β and β_{int} are identifiable as long as the ρ values differ among the IV-to-outcome GWAS samples (see the Supplementary Material for more details)."

in the Section "int2MR: An integrative MR for detecting exposure-by-group interaction effects with group-separated GWAS summary statistics" of the main text (see Page 6, Lines 125-127).

The current model assumptions indeed imply that the group-specific genetic effect on the outcome is fully mediated by the effect of the exposure on the outcome. There is no additional direct group-specific genetic effect on the outcome. In other words, our current model does not allow for a separate group-specific genetic effect on the exposure beyond the common β, and the differential effect across groups is entirely captured through the interaction effect β_{int}.

We recognize that heterogeneous IV–to–exposure effects across groups would necessitate an extension of the model. That said, the commonly imposed assumption of homogeneous IV–to–exposure effects is essential to guarantee the identifiability of β and β_{int}.

b) How is the model distinguish $\Gamma_j = \beta\gamma_j + \alpha_j^*$, where $\alpha_j^* = \beta_{int}\gamma_j + \alpha_j$ (I assume this might also be related to the assumption of uncorrelated balanced pleiotropy)? An explicit and detailed description of model assumptions will be very helpful.

Response to Comment 2b): Our model distinguishes $\Gamma_j = \beta\gamma_j + \alpha_j^*$, where $\alpha_j^* = \beta_{\text{int}}\gamma_j + \alpha_j$, by leveraging a set of key assumptions. We added following information in the Subsection "int2MR: An integrative MR for detecting exposure-by-group interaction effects with group-seperated GWAS summary statistics" (see Page 4, Lines 28-35):

"Our framework assumes that IVs must satisfy the standard core assumptions: (1) Relevance: the IVs are associated with the exposure (i.e., the IV-to-exposure effects, γ_j, are nonzero and assumed homogeneous across groups), (2) Independence: the IVs are independent of any unmeasured confounders, and (3) Exclusion Restriction: the IVs affect the outcome only through their effects on the exposure. We relax this last assumption by allowing for uncorrelated pleiotropy. We assume balanced pleotropy, α_j with $\mathbb{E}(\alpha_j) = 0$. That is, any residual direct effects of the IV on the outcome are assumed to average out. In our model, a “valid” IV is one that meets these conditions."

We clarify that our model distinguishes the total IV-to-outcome effect as $\Gamma_j = \beta\gamma_j + \alpha_j^*$, where $\alpha_j^* = \beta_{\text{int}}\gamma_j + \alpha_j$. This decomposition relies on a set of key assumptions that enable

identification of the interaction effect β_{int} separately from the main causal effect β.

First, we assume that the direct pleiotropic effects α_j are independent of the IV-to-exposure effects γ_j and are balanced, meaning $\text{E}(\alpha_j) = 0$. Under this assumption, any systematic association between the instruments and the outcome that scales with γ_j cannot be attributed to pleiotropy, and is instead interpreted as arising from the interaction effect β_{int}.

Second, we assume that the IV-to-exposure associations γ_j are homogeneous across groups. This ensures that group-specific differences in the IV-to-outcome associations are attributable to the interaction effect, rather than heterogeneity in the IV-to-exposure relationships.

Together, these assumptions allow the model to leverage variation in γ_j across SNPs to separate the main causal effect (β) and the interaction effect (β_{int}) from the pleiotropic effect.

c) Based on the current model $\Gamma_j = (\beta + \beta_{int} \cdot \rho) \cdot \gamma_j + \alpha_j$, is it possible to infer parameters of interest using only multiple combined outcome GWAS dataset with known ρ's?

Response to Comment 2c):

Yes, it is possible to infer the parameters of interest using only multiple combined outcome GWAS datasets, provided that the proportions (ρ's) differ across datasets. The variation in ρ across studies enables us to disentangle the main causal effect (β) from the interaction effect (β_{int}). For a formal demonstration, we have added a proof of model identifiability in the Subsection "Model Idenitifiability" in the Supplementary Material. In the main text, we mentioned that "β and β_{int} are identifiable as long as the ρ values differ among IV-to-outcome GWAS samples (see the Supplementary Material for more details)." (see Page 6, Lines 125-127).

Comment 3: *How is the power of testing β and β_{int} related to the sample sizes of different GWAS datasets? If this cannot be derived analytically, a more extensive simulation studies will be helpful.*

Response to Comment 3:

The power to detect both the main causal effect (β) and the interaction effect (β_{int}) is closely tied to the sample sizes of the contributing GWAS datasets. Larger sample sizes lead to

smaller standard errors for the IV-to-exposure and IV-to-outcome effect estimates, thereby enhancing the precision and increasing power to detect true associations.

This relationship is particularly important when incorporating a group-combined GWAS, which often has a substantially larger sample size than the group-stratified datasets. In such cases, the inclusion of the combined dataset can substantially improve the power for detecting β_{int}, as it provides more stable estimates for the average causal effect across groups.

To support this, we have included an analytical derivation of the conditional posterior variance in Supplementary Subsection "Posterior Distributionand Benefits of Data Integration" . There (see Page 15), we show that the marginal posterior variances of β and β_{int} conditioning on the true IV-to-exposure effects $\{\gamma_j\}$'s correspond to the diagonal entries of the inverse precision matrix:

$$I = \sum_{j=1}^{p} \begin{pmatrix} \frac{\gamma_j^2}{\tilde{s}_{0,j}^2} + \frac{\gamma_j^2}{\tilde{s}_{1,j}^2} + \frac{\gamma_j^2}{\tilde{s}_{2,j}^2} & \cdots \\ \cdots & \frac{\gamma_j^2}{\tilde{s}_{1,j}^2} + \rho^2 \frac{\gamma_j^2}{\tilde{s}_{2,j}^2} \end{pmatrix},$$

where each $\tilde{s}_{k,j}^2 = \widehat{s}_{k,j}^2 + \widetilde{\sigma}_{\alpha_k}^2$ includes both the estimated sampling variance and the prior variance of pleiotropic effects. Notably, the term $\tilde{s}_{k,j}^2$ scales approximately as $1/n_{Y_k}$, indicating that increases in sample size n_{Y_k} directly reduce posterior uncertainty.

Comment 4: *For the simulation results, besides Type-I error and power, could the authors also show the averaged estimates, MSE, bias etc. (at least in the supplementary)*

Response to Comment 4: We have added Table S1 in the Supplementary Material (see Page 6) to provide the RMSE of our method and competing methods.

Comment 5: *In simulation, could the authors also consider scenarios with directional pleiotropy and/or correlated pleiotropy? It will be interesting to see how robust the model is in these scenarios.*

Response to Comment 5: We agree that examining the model's robustness under directional and correlated pleiotropy is important. In our current simulation studies, we focused on scenarios with uncorrelated balanced pleiotropy because our model's identifiability relies on the assumption that any pleiotropic effects (α_j) are independent of the IV-to-exposure effects (γ_j) and average to zero.

In the existing MR methods, it is commonly assumed in the literature that the pleiotropic effect is uncorrelated with the exposure. We followed this assumption in our study.

When correlated pleiotropy is present, the situation becomes more complex, requiring the development of a new method. We added a discussion on the limitations of our method in Section "Discussion" in the main text as follows (see Page 14, Lines 355-359):

"In particular, if the correlated pleiotropic effect differs among the GWAS samples, the resulting pattern may confound the interaction effect (β_{int}) with the pleiotropic effect. In these cases, it is difficult to disentangle whether the observed deviations in the IV–outcome associations are due to a true interaction or to systematic pleiotropic biases."

One potential approach to tackle this issue is to conduct sensitivity analyses or to pre-screen IVs for evidence of correlated pleiotropy, excluding those that show strong directional effects. We acknowledge that extending our framework to directly model such pleiotropic structures is technically challenging and remains an important area for future research.

Comment 6: *In real data application, what are the results for shared effects β? Could the authors also present some results only based on the combined GWAS dataset using existing MR methods, and discuss the comparison?*

Response to Comment 6: We added results only based on the combined GWAS dataset using existing MR methods. Specifically, we added the heatmap of the group-specific effects β and $\beta + \beta_{\text{int}}$ to the Supplementary Material as Figure S1 (see Page 9). The heatmap shows that the inflammation and immune-related risk factors have smaller effect size in the oldest old, suggesting that chronic inflammation is reduced in the oldest-old group.

However, detecting the interaction effects directly based on the combined GWAS dataset is not feasible. Existing MR methods can not distinguish the shared effect β from the combined effects $\beta + \rho \cdot \beta_{\text{int}}$. Therefore, existing MR methods cannot be applied to directly identify the interaction effects.

Comment 7: *Lines 234 mentioned 35 exposures are examined, but line 242 mentioned "the analysis was restricted to 51 exposures". And Fig4 show neither 35 nor 51 exposures. Which one is correct?*

Response to Comment 7: We are sorry for the confusion. We re-ran the analyses for the

Table 1: **Risk exposures and their age-group interaction effect estimates obtained by int2MR for clinically diagnosed AD**

**Exposure**	**#IVs**	$\widehat{\beta}_{\textbf{int}}$	***p*-value**	**FDR adj.**
Fluid Intelligence Score	172	1.592	1.83×10^{-8}	9.52×10^{-7}
Atrial Fibrillation	418	0.602	2.73×10^{-6}	7.09×10^{-5}
Systemic Lupus Erythematosus	214	0.186	4.97×10^{-5}	0.000646
Neuroticism	251	0.975	6.48×10^{-5}	0.000674
Self-Reported Hypertension	666	2.400	0.000118	0.001022
Crohn's Disease	175	0.249	0.000276	0.002050
Eosinophil Count	836	-0.657	0.001201	0.006942
Sum of Eosinophil and Basophil Counts	775	-0.720	0.001196	0.006942
Inflammatory Bowel Disease	231	0.284	0.002026	0.010535
Self-Reported Eczema or Dermatitis	75	5.342	0.003096	0.014629
Self-Reported Hypothyroidism	419	2.643	0.004504	0.019517
Hair or Balding Pattern 4	601	-1.001	0.005527	0.022107
Coronary Artery Disease	97	0.751	0.009682	0.034958
Rheumatoid Arthritis	443	0.155	0.010084	0.034958

Significant exposure-by-age-group interactions for clinically diagnosed Alzheimer's disease (AD) identified using the int2MR method. Displayed are the interaction effect estimates $\widehat{\beta}_{\text{int}}$, p-values, and FDR-adjusted p-values.

two diseases using the same standard GWAS significance threshold ($p < 5 \times 10^{-8}$), and the same LD-clumping threshold ($r^2 < 0.05$). We eaxmined 51 exposures in total.

The full list of 51 exposures examined in our study can be found in Supplementary Material Table S2.

Comment 8: *What is the result of clinically diagnosed AD?*

For clinically-diagnosed Alzheimer's disease, our int2MR analysis identified fourteen exposures with significant exposure-by-age-group interaction effects. Full results are summarized in Table S5 in Supplementary Material S1 Appendix (see Page 12). We also include our results here in Table 1

Similar to the pathology-based analysis, our findings highlight that both cognitive-reserve and immune-related pathways exhibit significant interaction effects on clinically diagnosed AD.

Comment 9: *The current heatmap only shows the difference in effect, could the authors also present a heatmap comparing the total effects in the two groups? This may give more information than only presenting the difference.*

Response to Comment 9: We have now added an additional heatmap (Figure S1) in the Supplementary Material S1 Appendix (see Page 8) that displays the Z-scores of shared effect (β) and total effects ($\beta + \beta_{\text{int}}$), corresponding to the group specific effects for both groups as estimated by the int2MR method. We hope this supplementary figure provides a more comprehensive view of the group-specific risk exposures alongside the differences previously presented.

Point-by-Point Response for Reviewer 2

Comment 1: *In models (1-3), it is unclear how the authors handle (γ_j). Is it treated as a random variable with a prior, or as a fixed but unknown parameter?*

Response to Comment 1:

In the Section "Modeling interaction effects in the group-combined GWAS: from individual-level data to GWAS summary statistics" (see), we claified that in our model, the IV-to-exposure effect $\{\gamma_j\}_{j=1}^{p}$is treated as a latent random variable rather than a fixed parameter (see Page 5, Lines 78). Specifically, we assign it a normal prior distribution, i.e., $\gamma_j \sim \mathcal{N}\left(0, \sigma_\gamma^2\right)$. This approach reflects the assumption that the true IV-to-exposure effects are centered around zero with variability captured by σ_γ^2, and it allows us to model the uncertainty and variability across different SNPs in a Bayesian framework.

Comment 2: *The authors state that "int2MR improved power by jointly testing for main and interaction effects." This implies that the main effect should be non-zero. Two clarifications are needed: What if the main effect is zero? Additionally, can the method focus solely on testing the interaction effect?*

Response to Comment 2: Our framework is flexible: Even if the true main effect is zero, the joint model can still yield power for detecting the interaction effect through differences between groups. In scenarios where the main effect is zero, the power for detecting the interaction effect will naturally depend on the variability and strength of the group-specific differences.

Comment 3: *In the real data analysis, how did the authors specify (ρ_k)? Is it known or unknown in practice?*

Response to Comment 3: In Section "Flexible int2MR allowing the integration of group-specific and group-combined GWAS statistics" (see Page 5, Lines 93-94), we clarify that in our real data analysis, the proportion parameter (ρ_k) is treated as known. It can be interpreted as a summary statistics of the group label of each individual. For example, when analyzing mixed-group GWAS data, the value of ρ_k is determined from the sample composition reported by the study (e.g., the proportion of females in a sex-combined analysis). This information

is typically available from the GWAS study metadata or design, enabling us to incorporate it directly into our model.

Comment 4: *Regarding sensitivity analysis, the authors used thresholds of* (5×10^{-8}) *(ADHD) or* (10^{-8}) *(Alzheimer's Disease) to select instrumental variables (IVs) and then performed LD clumping with an* (r^2) *threshold of 0.05, assuming all IVs are valid. Please conduct sensitivity analyses to ensure the robustness of the results.*

Response to Comment 4:

Thanks for pointing it out. To assess the robustness of our findings to different IV-selection criteria, we re-ran the analyses for the two diseases using the same standard GWAS significance threshold ($p < 5 \times 10^{-8}$), and the same LD-clumping threshold ($r^2 < 0.05$).

We also run the analyses under a more stringent LD clumping threshold ($r^2 < 0.01$). The corresponding results remain close to those using the original threshold ($r^2 < 0.05$). To demonstrate our results, we added Table S3 (see Page 16) and Table S4 (see Page 17) in the Supplementary Material S1 Appendix for the comparison of the real data analysis on ADHD and Alzheimer's disease respectively, under two LD clumping thresholds. In addition, since Alzheimer's disease has 6 outcomes, we added Figure S2 (see Page 9) for the comparison of results under two LD clumping thresholds, corresponding to the Figure 4 in the main text. The results are similar with different thresholds.

Point-by-Point Response for Reviewer 3

Comment 1: *Both the analytical derivation and simulations focus on linear regression models, yet both both ADHD and Alzheimer's disease are binary outcomes, as it seems. It is not clear if the proposed methods extend to generalized linear models. For example, does the assumed linear relationship with rho still hold? In addition, the exposure X is treated as linear, but some exposures in the real data may not be. The authors should clarify these points, possibly with additional simulations that mimic the real data, and discuss potential limitations.*

Response to Comment 1:

Our primary focus on linear regression models was motivated by both simplicity and convention in summary-statistics-based MR analyses. In practice, the summary statistics for binary outcomes are typically derived from logistic regression models, and the log-odds ratios can be treated as approximately linear in our framework, provided that appropriate transformations are applied. We added the following paragraph to the Section "Discussion" (see Page 14, Lines 362-366):

"Our current work focuses on linear models. In many cases, especially when effect sizes are modest, the linear approximation provides a reasonable first-order approximation for binary outcomes. We plan to incorporate binary outcomes and nonlinear relationships in our future studies and refine the robustness and applicability of our methods under these conditions."

Comment 2: *The simulation setup includes U_{Y0} or U_{Y1} as (unobserved) confounding variables, but their role as confounders is unclear since they do not appear to be correlated with X. A clearer description or modification of data generation is needed.*

Response to Comment 2:

We have revised our simulation setup to better clarify the role of confounders. We updated the description of simulation design in the Supplementary Material S1 Appendix (see Page 4, Lines 67-74):

"In particular, we assume that the unobserved confounding effect on the exposure is represented by U_X, which is generated as a standard normal variable. U_{Y_0} and U_{Y_1} are assumed to consist of independent and identically distributed (i.i.d.) normal entries with standard

deviations U_0 and U_1, respectively. To ensure that the outcome confounders are related to the exposure, we model U_{Y_0} and U_{Y_1} as direct linear functions of U_X. Specifically, we define:

$$U_{Y_0} = U_0 \cdot U_X$$
$$U_{Y_1} = U_1 \cdot U_X$$

Here, U_0 and U_1 denote the standard deviations for the confounding effects in groups 0 and 1 , respectively. This formulation guarantees that the confounders U_{Y_0} and U_{Y_1} are correlated with X via U_X."

We have updated the simulation section to reflect these modifications.

Comment 3: *Comparison with competing methods.*

a) For the 2SLS method, why is the IV used for sex as well (in addition to IV for exposure)?

Response to Comment 3a):

Thanks for pointing it out. We re-did the simulation studies in which sex (and more generally, the group label) was treated as a covariate (not as an exposure with instrument variables) in the second-stage linear regression and did not require an instrument on its own. We have modified our manuscript accordingly to reflect this correction.

We have added a paragraph in the Subsection "Comparison with existing methods" in the Supplementary Material S1 Appendix as follows (see Pages 2 and 3, Lines 37-43):

"Methods that leverage individual-level data facilitate direct modeling of covariates and their interactions. In our analysis, we compared our approach with two standard methods:

1. Two-Stage Least Squares (2SLS):

In the 2SLS framework [1, 2], the procedure is carried out in two steps. First, the exposure X is predicted from the instrumental variables $G_{i\,i=1}^{\,p}$:

$$\text{First-stage regression: } \widehat{X} = \sum_{i=1}^{p} \widehat{\gamma}_i G_i = \mathbb{E}\left(X \mid \{G_i\}_{i=1}^{p}\right)$$

Next, the outcome Y is regressed on the predicted exposure $\widehat{X}$, the group variable S (e.g.,

sex or age group), and their interaction:

$$\text{Second-stage regression: } \mathbb{E}\left(Y|\widehat{X}, S\right) = \widehat{\beta}\widehat{X} + \beta_S S + \widehat{\beta}_{\text{int}}\widehat{X} \circ S,$$

This two-step procedure allows for the estimation of both the main effect of X and the interaction effect between X and S."

b) In the OLS model, it seems that the data were generated to have a differential G effect by group, but yet this effect was not included in the model. It is not clear then if the observed type I error inflation for OLS is due to omission of G (i.e., model misspecification) or unmeasured confounding, which is the purpose of a MR analysis.

Response to Comment 3b): In our OLS analysis, we used individual-level data and included the group label as a covariate. We included the following information in the Subsection "Comparison with existing methods" in the Supplementary Material S1 Appendix as follows (see Pages 3, Lines 44-46):

"We also compared our method with a conventional linear regression model that directly incorporates the exposure, the covariate group, and their interaction:

$$\mathbb{E}\left(Y|X, S\right) = \beta \cdot X + \beta_S \cdot S + \beta_{\text{int}} X \circ S."$$

As a result, the observed type I error inflation in the OLS model is likely due to unmeasured confounding.

c) In the Introduction, the authors describe several individual-level-data methods for testing interaction but did not include them in comparisons. Why were these omitted in favor of MR methods or 2SLS/OLS? A discussion on this choice or inclusion of these methods for comparison that more target interaction would be helpful.

Response to Comment 3c): We appreciate the reviewer's comment on this point. Recently, Zhu el al. (2024) [2] developed a powerful approach to identify gene-environment interactions but it cannot be straightforwardly repurposed for estimating exposure–covariate interaction effects in an MR framework. Our focus in the manuscript is on developing a method that

leverages summary statistics, which are widely available for assessing interaction effects. We selected two-stage least squares (2SLS) and ordinary least squares (OLS), both being widely used individual-level data methods, as benchmarks to evaluate our approach when individual-level data are available. In the "Introduction" and "Discussion" Sections in the manuscript, we would like to clarify that int2MR was developed to meet the needs of settings where only summary statistics are available and interaction methods requiring individual-level data cannot be applied.

d) It seems to me that inclusion of conventional MR methods is distractive, given the focus is on interaction and the real data analysis is also on interaction.

Response to Comment 3d): We agree that the focus of our work is on interaction effects, and that conventional summary-statistics-based MR methods cannot be directly applied to detect the interaction effects. However, we included these methods to provide a broader context and to provide the power comparison in detecting risk factors (main or main+interaction) in the presence of group-specific effects when group-specific GWAS data is available.

Comment 4: *In the simulation for the power comparison, it is interesting and surprising that int2MR has comparable power to OLS. How much of the variation in X is explained by all the IVs together? Is this common in the real data situation? In addition, the integration of an additional group-combined GWAS improves power for int2MR. Do other methods in the comparison have similar effective sample sizes?*

Response to Comment 4: In our simulations, we add the following information into the Subsection "Data Generation" in the Supplementary Material S1 Appendix: "The variance of instruments together explains 37% of the variance of exposure on average in power simulation." (see Page 5, Lines 93-94)

While this level of variance explained may exceed what is typically observed in empirical data, it is representative of highly heritable traits and allows us to assess the upper-bound performance of the method when strong instruments are available.

In our int2MR method, integration of additional group-combined GWAS data substantially increases the sample size and thereby boosts the power, which is comparable or even higher

than that of OLS. OLS relies solely on the available group-specific data (with a 2000-sample size of the reference group and a 1000-sample size of the comparison group). Without integrating the additional group-combined GWAS dataset, OLS has higher power than int2MR in detecting both the main effect and the shared effect. In contrast, int2MR leverages the extra information provided by the group-combined data, which is not utilized by other individual-level MR methods.

Comment 5: *On page 6, the authors state that int2MR may not be suitable when IVs are highly correlated. Could they clarify why and demonstrate, via simulations, the extent to which IV correlation affects the method's performance?*

Response to Comment 5: The int2MR framework relies on the assumption that the selected IVs are either independent or only weakly to moderately correlated. We added the following information in Subsection "Allowing correlated SNPs as IVs by accounting for linkage disequilibrium (LD)" (see Page 7, Lines 156-158):

"In this study, we apply LD clumping to select a set of relatively independent instruments using a threshold of $r^2 < 0.05$."

Moreover, in the Supplementary Material S1 Appendix (Subsections “Additional Results on Data Analysis: Identifying Risk Factors with Sex-Biased Effects on ADHD” and “Additional Results on Data Analysis: Identifying Age-Group-Specific Risk Factors for Alzheimer’s Disease in the Oldest-Old”), we now present a sensitivity analysis in which we compare our primary LD threshold ($r^2 < 0.05$) against a more stringent threshold ($r^2 < 0.01$). The results remain largely unchanged under these two LD clumping thresholds.

Comment 6: *Since the proposed method is Bayesian, it would be useful to assess how robust the proposed test statistics is against the misspecification of prior distributions. A discussion or sensitivity analysis for the simulated and particularly the real data would strengthen the conclusions.*

Response to Comment 6: To address this issue, we performed simulation studies under three near-noninformative inverse–gamma priors. We added a supplementary Section "Sensitivity to Inverse–Gamma Hyperparameters" in the Supplmentary Material to report the results. With different prior settings, our Bayesian int2MR test maintains type I error closer

to the nominal level than both standard alternatives (for details, see Table S2).

In Table S2 (see Page 6), we report the type I error rates (nominal 5%) for testing the interaction effect $H_0 : \beta_{\text{int}} = 0$ and testing the shared causal effect $H_0 : \beta = 0$ under each prior, alongside corresponding 2SLS and OLS error rates across different scenarios.

References

[1] Basmann RL. A generalized classical method of linear estimation of coefficients in a structural equation. Econometrica: Journal of the Econometric Society. 1957; p. 77–83.

[2] Zhu X, Yang Y, Lorincz-Comi N, Li G, Bentley AR, de Vries PS, et al. An approach to identify gene-environment interactions and reveal new biological insight in complex traits. Nature Communications. 2024;15(1):3385.
