# Supplementary material for: Integrative Mendelian randomization for detecting exposure-by-group interactions using group-specific and combined summary statistics
Source: PLoS Genet. 2025 Sep 11;21(9):e1011819. doi: 10.1371/journal.pgen.1011819 (PMC12440225; doi:10.1371/journal.pgen.1011819)
Supplement: Attachment — Submitted filename: response_r2.pdf [file pgen.1011819.s006.pdf]

From: Lin S. Chen

Department of Public Health Sciences

The University of Chicago

Chicago, Illinois 60637

Jul 21st, 2025

Dear Editorial Board of *PLoS Genetics*,

We are writing to submit the revised manuscript entitled "Integrative Mendelian randomization for detecting exposure-by-group interactions using group-specific and combined summary statistics".

We would like to thank the editor and three reviewers for their thorough and constructive comments and suggestions. We have carefully addressed each reviewer's comments in a comprehensive, point-by-point manner, as outlined in the accompanying document. The reviewer's comments are presented in italic, followed by our detailed responses. We have revised the manuscript and Supplementary Material S1 Appendix accordingly.

In response to the question, "Have all data underlying the figures and results presented in the manuscript been provided?", we have provided the GWAS summary statistics for the IV-to-outcome effects derived from the Religious Orders Study and the Rush Memory and Aging Project (ROSMAP), along with all primary results underlying our analyses at `https://doi.org/10.5281/zenodo.16341091`. All the GWAS summary statistics of IV-to-exposure effects used in the manuscript are publicly available. Related links to the summary statistics can be found in Supplementary Material S2 Table.

Thank you for your consideration. We would be happy to provide any further information or clarification as needed.

Sincerely yours,

Lin S. Chen and co-authors

Point-by-Point Response for Reviewer 1 (Major changes made in the main text are highlighted in yellow)

Comment 1: *The typo in the equation below line 71 appears to remain uncorrected and is the same as in the previous version.*

Response to Comment 1:

We apologize for the negligence. We have corrected the equation below line 73 in the revised manuscript.

Comment 2: *Regarding Comment 6: I did not see the inclusion of results based on the combined GWAS dataset using existing MR methods. In addition, could the authors also perform traditional MR analyses using the sex-stratified GWAS data and compare those results with the findings presented in Figure S1?*

Response to Comment 2:

We have updated the Supplementary Material to include two additional sets of MR analyses using existing methods, IVW and MR-Egger, applied to GWAS summary statistics from the full population (combining both age groups).

The reviewer requested sex-stratified analyses, but we believe that the reviewer means age-group-stratified GWAS data. Our study focuses on age-group interaction effects (death-age 95+ vs 95-), and sex-stratified summary statistics are not available for this dataset. We have summary statistics for the oldest-old group (death-age 95+) and for the full population, but not for the younger group alone (death-age 95-).

Therefore, we applied IVW and MR-Egger to both the full dataset and the oldest-old subgroup. These methods cannot estimate effects in the younger group directly, as we lack summary statistics for that group. However, our proposed method, int2MR, leverages both the full-population and oldest-old data to detect group-specific effects, including those in the younger group (death-age 95-). We have included the following results and explanation in Supplementary Material S1 (Page 10).

"We applied two conventional Mendelian randomization methods, IVW and MR-Egger, to the full-population GWAS summary statistics in order to estimate the overall exposure effects on

Alzheimer's disease. The resulting Z-scores are displayed in Figure S3. When compared with the int2MR-derived Z-scores (Figures S1(a) and (b)), we observe a strong concordance, particularly with the *younger (95-) subgroup* (Figure S1(b)). This alignment likely reflects the fact that the oldest-old group represents only a small fraction (408/2587) of the full sample, causing traditional MR estimates in the overall cohort to be driven predominantly by the larger, younger majority.

Similarly, we applied IVW and MR–Egger solely to the oldest-old GWAS summary statistics to estimate exposure effects on Alzheimer's disease (AD) within this subgroup. The resulting Z-scores are presented in Figure S4. When these traditional MR heatmaps are compared with those obtained from our int2MR method, which integrates GWAS summary statistics from both the oldest-old group and the full population (Figure S1(a)), the Z-score distributions across exposures remain largely consistent."

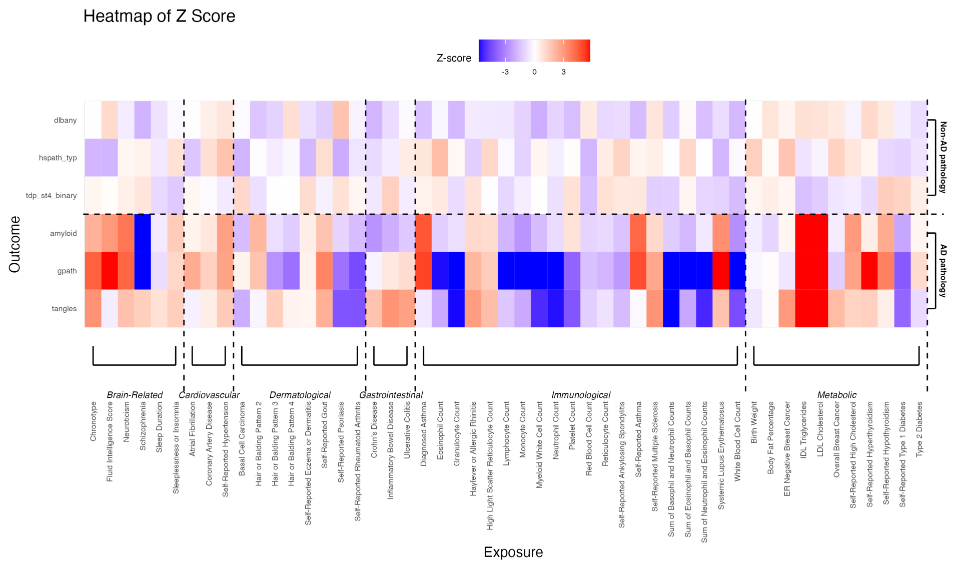

(a)

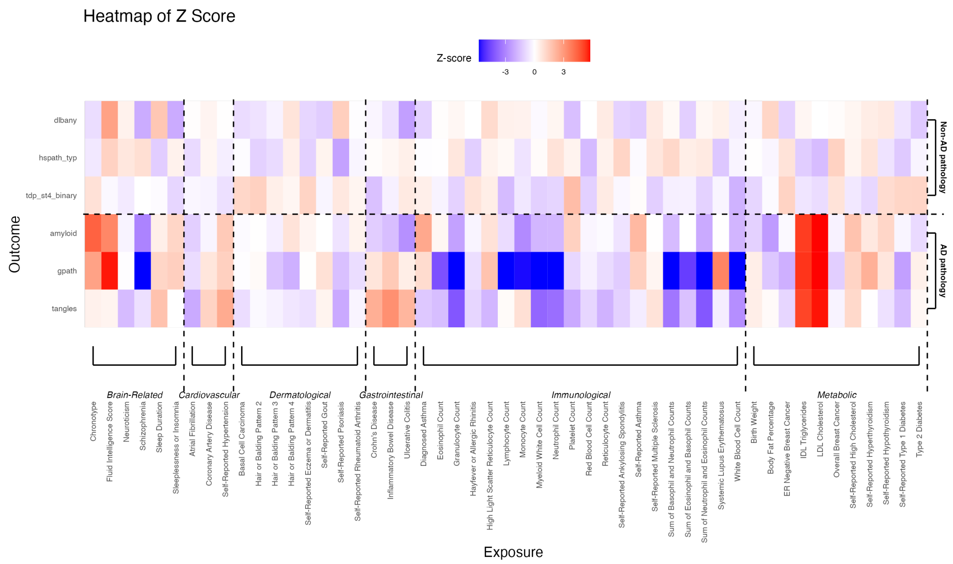

(b)

Figure S3: **Two heatmaps showing the Z-score of the total effects on AD pathologies (bottom panel) and non-AD pathologies (top panel) based solely on the GWAS summary statistics for the full population.** Here, the figure (a) shows these Z-scores estimated by IVW, while the figure (b) shows them estimated by MR-Egger.

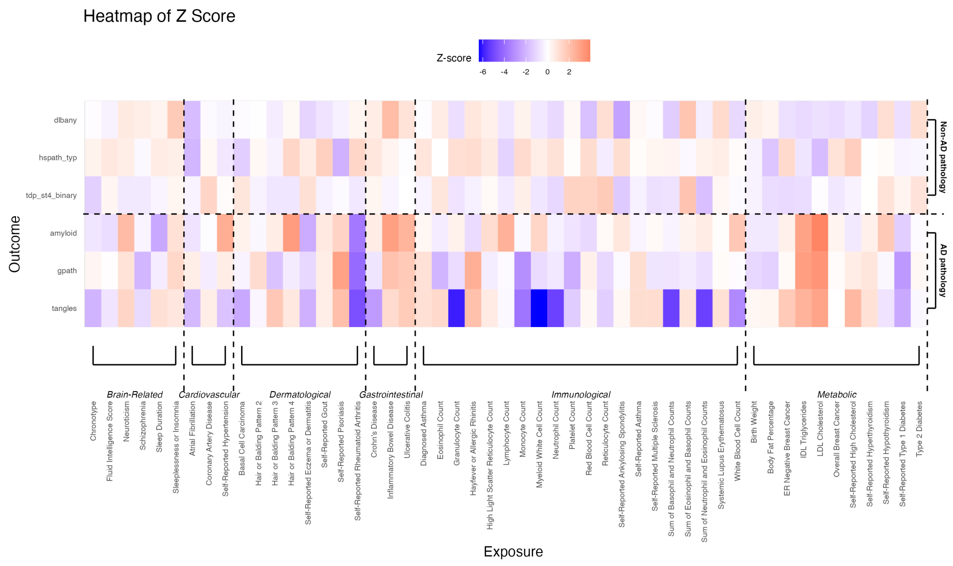

(a)

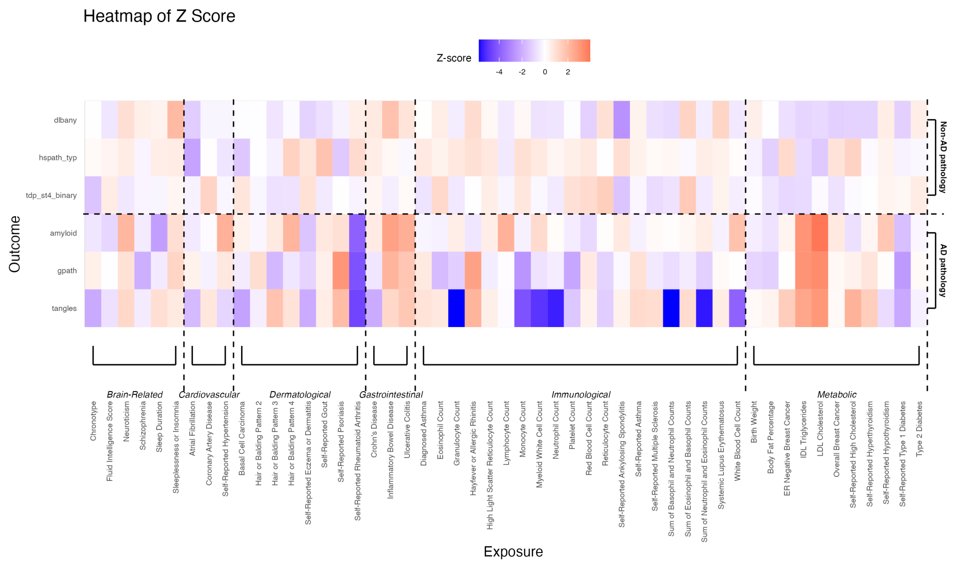

(b)

Figure S4: **Two heatmaps showing the Z-score of oldest-old group-specific effects on AD pathologies (bottom panel) and non-AD pathologies (top panel) based solely on the GWAS summary statistics for the oldest-old group.** Here, the figure (a) shows these Z-scores estimated by IVW, while the figure (b) shows them estimated by MR-Egger.

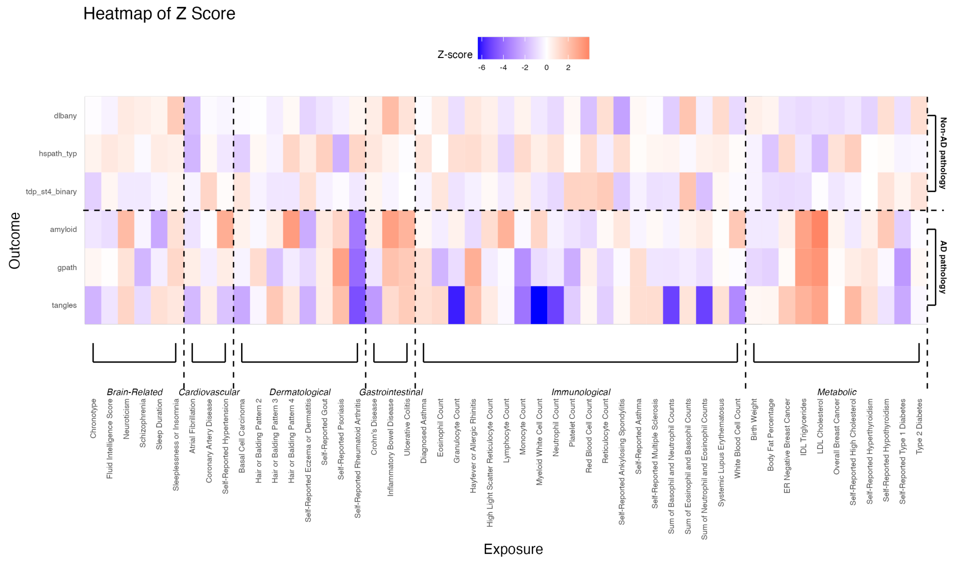

(a)

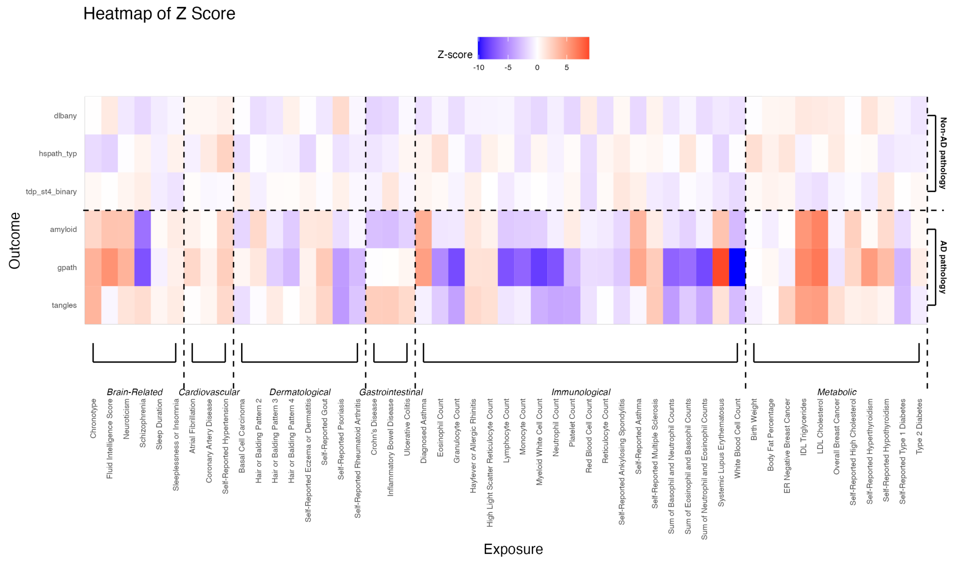

(b)

Figure S1: **Two heatmaps showing the Z-scores of the age-group specific effects on AD pathologies (bottom panel) and non-AD pathologies in the oldest-old group versus the younger age group.** Here, figure (a) shows the age-group specific effects in the oldest-old group (95+) while figure (b) shows the age-group specific effects in the younger age groups (95-).

Point-by-Point Response for Reviewer 3

Comment 1: *1. The response to comment is superficial. The data analysis used binary outcomes, and yet the proposed method is developed only for linear regression. This mismatch is unacceptable and must be rectified.*

Response to Comment 1:

We would like to first clarify that the effect estimates from MR analysis for binary outcomes can be interpreted as a log linear approximation. In practice, many large-scale GWAS of disease endpoints are conducted via logistic regression, so the published summary statistic is

$$\beta_{\text{logit}} = \log(\text{OR})$$

which is itself a linear coefficient on the log-odds scale. For low-prevalence binary outcome (such as prevalence $p < 0.1$), a first-order Taylor expansion of the logistic link shows

$$\beta_{\text{lin}} \approx \beta_{\text{logit}} \quad \text{up to } O(p),$$

where β_{lin} is the slope from an idealized linear regression of a "pseudo-continuous" outcome. Concretely, if a GWAS reports an odds ratio $\widehat{\text{OR}}$ with standard error SE_{OR}, we set

$$\widehat{\beta}_{\text{pseudo}} = \log(\widehat{\text{OR}}), \quad \text{SE}_{\text{pseudo}} = \frac{\text{SE}_{\text{OR}}}{\widehat{\text{OR}}}$$

by the delta method, and then apply our linear-regression bias-correction using $\widehat{\beta}_{\text{pseudo}}$ and $\text{SE}_{\text{pseudo}}$.

Ideally, the summary statistics should be re-calculated on the linear scale, but in majority of the cases, researchers do not have access to the raw data. Using summary statistics from existing large consortia and combined with *log linear approximation*, we can get valid MR inference (testing) and also the correct effect direction, as well as relative strengths of effects.

Indeed, this represents a constraint, and it also reflects a broader challenge and limitation in the field of MR analysis. In the existing literature, log-linear models are often used to test effects. Theoretical work is frequently conducted under the assumption of linear models for

continuous outcomes [Greenland and Thomas, 1982, Robbins et al., 2002], with extensions to binary outcomes. In many cases, particularly when effect sizes are modest, such linear approximations are considered reasonable for binary outcomes. We kindly request the reviewers and editors do not penalize the current work on common challenges in the field and value the novel insights and contributions of our work in evaluating interaction effects.

Comment 2: *The rationale for omitting Zhu et al. (2024) for comparison is unpersuasive. By the same logic, OLS—also not designed for MR—should have been excluded.*

Response to Comment 2: We would like to clarify that our proposed int2MR method and the method by Zhu et al. (2024) were developed for different purposes and are intended for distinct applications. Zhu et al. (2024) examines SNP-by-covariate interactions on exposure, i.e., SNP x covariate → exposure → outcome. Our method studies exposure-by-covariate interactions on outcome, i.e., SNP → exposure x covariate → outcome.

For example, in our second real-data application (see Section "Data Analysis: Identifying Age-Group-Specific Risk Factors for Alzheimer's Disease in the Oldest-Old," p. 10), the data include: 1) GWAS summary statistics for AD from the ROSMAP study, including two age groups (death-age 95+ vs 95-), and 2) GWAS summary statistics for a set of risk (exposure) factor (e.g., asthma). We can apply our int2MR method to detect interaction effects between the exposure asthma and age-group on AD. For this data, the method of Zhu et al. can be applied to detect SNP by age-group interactions on asthema or AD, but cannot detect the interaction between asthma and age-group.

Given the differences in the input data and resulting outputs/conclusions, we respectfully chose not to include the method of Zhu et al. (2024) in the comparison with our int2MR approach in the manuscript.

In summary, the method by Zhu et al. primarily focuses on SNP-by-covariate interactions affecting the exposure and then possible outcome, while our method int2MR targets exposure-by-covariate interactions on the outcome. See Figure R1) for an example.

(a)

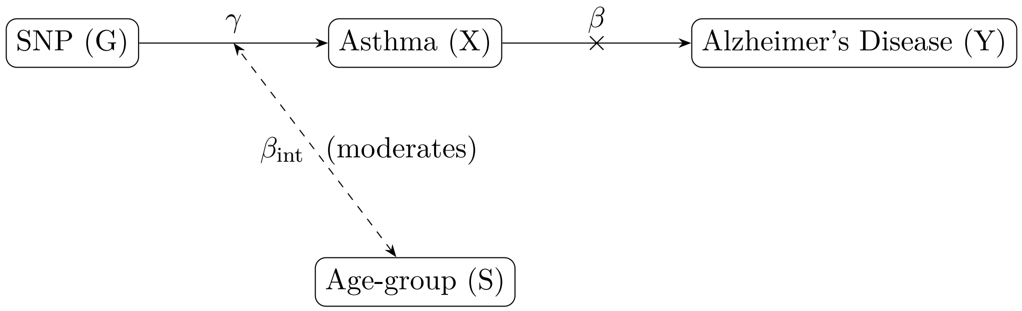

(a) Testing SNP × Age-group Interaction [Zhu et al., 2024]

(b)

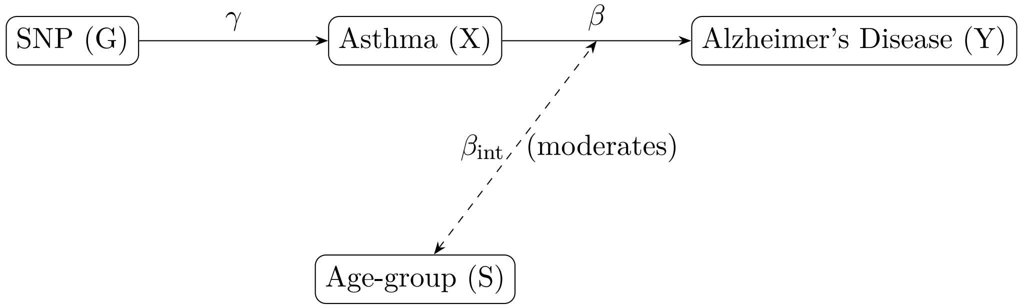

(b) Testing Asthma × Age-group Interaction (int2MR)

Figure R1: An illustration of the difference between our proposed int2MR method and MR-GxE method proposed in Zhu et al. [2024]: (a) Zhu et al. [2024] focuses on SNP-by-covariate interactions on exposure; (b) our int2MR aims to detect exposure-by-covariate interactions.

Comment 3: *The response states that "relatively independent" SNPs are required, yet the manuscript claims the method tolerates "moderate" correlation. Clarify what constitutes moderate correlation and soften the wording of generalization if necessary.*

Response to Comment 3:

Sorry for the confusion. The model was developed to allow moderately correlated SNPs as IVs by accounting for LD structures. In the data analysis, we applied the stringent LD clumping threshold to select the relatively independent SNPs so that we do not need to consider LD. We agree that this is unclear. We deleted the sentence "the method tolerates moderate correlation" and added the following to page 10 to avoid confusion: "In this analysis, we apply LD clumping to select a set of relatively independent IVs without considering their LD structures".

Comment 4: *The explanation about power comparison in the response is strong, but the key points need to be integrated into the main text to prevent misinterpretation and ensure balanced representation.*

Response to Comment 4:

We added the following content to the Section Power Comparison (See Page 8 of the main text, highlighted in yellow):

"In our int2MR method, integration of additional group-combined GWAS data substantially increases the sample size and thereby boosts the power, which is comparable or even higher than that of OLS. OLS relies solely on the available group-specific data (with a 2000-sample size of the reference group and a 1000-sample size of the comparison group). Without integrating the additional group-combined GWAS dataset, OLS has higher power than int2MR in detecting both the main effect and the shared effect. In contrast, int2MR leverages the extra information provided by the group-combined data, which is not utilized by other individual-level MR methods."

References

Sander Greenland and Duncan C Thomas. On the need for the rare disease assumption in case-control studies. *American journal of epidemiology*, 116(3):547–553, 1982. https://doi.org/10.1093/oxfordjournals.aje.a113439.

Anthony S Robbins, Susan Y Chao, and Vincent P Fonseca. What's the relative risk? a method to directly estimate risk ratios in cohort studies of common outcomes. *Annals of epidemiology*, 12(7):452–454, 2002. 10.1016/s1047-2797(01)00278-2.

Xiaofeng Zhu, Yihe Yang, Noah Lorincz-Comi, Gen Li, Amy R Bentley, Paul S de Vries, Michael Brown, Alanna C Morrison, Charles N Rotimi, W James Gauderman, et al. An approach to identify gene-environment interactions and reveal new biological insight in complex traits. *Nature Communications*, 15(1):3385, 2024. https://doi.org/10.1038/s41467-024-47806-3.
