# Supplementary material for: Integrative Mendelian randomization for detecting exposure-by-group interactions using group-specific and combined summary statistics
Source: PLoS Genet. 2025 Sep 11;21(9):e1011819. doi: 10.1371/journal.pgen.1011819 (PMC12440225; doi:10.1371/journal.pgen.1011819)
Supplement: Attachment — Submitted filename: Review.docx [file pgen.1011819.s004.docx]

The paper proposes an integrative Mendelian randomization (int2MR) for identifying interactions using GWAS summary statistics, either group-specific or combined with known group proportion. Simulation studies show that int2MR controls type I error. Two real datasets were analyzed for illustrating the method. The paper is well written, and the proposed method is a valuable addition to the MR toolbox, particularly for interaction analyses.

I have a few comments and suggestions for the authors to consider:

1. Both the analytical derivation and simulations focus on linear regression models, yet both both ADHD and Alzheimer’s disease are binary outcomes, as it seems. It is not clear if the proposed methods extend to generalized linear models. For example, does the assumed linear relationship with rho still hold? In addition, the exposure X is treated as linear, but some exposures in the real data may not be. The authors should clarify these points, possibly with additional simulations that mimic the real data, and discuss potential limitations.
2. The simulation setup includes U_Y0 or U_Y1 as (unobserved) confounding variables, but their role as confounders is unclear since they do not appear to be correlated with X. A clearer description or modification of data generation is needed.
3. Comparison with competing methods.
   - For the 2SLS method, why is the IV used for sex as well (in addition to IV for exposure)?
   - In the OLS model, it seems that the data were generated to have a differential G effect by group, but yet this effect was not included in the model. It is not clear then if the observed type I error inflation for OLS is due to omission of G (i.e., model misspecification) or unmeasured confounding, which is the purpose of a MR analysis.
   - In the Introduction, the authors describe several individual-level-data methods for testing interaction but did not include them in comparisons. Why were these omitted in favor of MR methods or 2SLS/OLS? A discussion on this choice or inclusion of these methods for comparison that more target interaction would be helpful.
   - It seems to me that inclusion of conventional MR methods is distractive, given the focus is on interaction and the real data analysis is also on interaction.
4. In the simulation for the power comparison, it is interesting and surprising that int2MR has comparable power to OLS. How much of the variation in X is explained by all the IVs together? Is this common in the real data situation? In addition, the integration of additional group-combined GWAS improves power for int2MR. Do other methods in the comparison have similar effective sample sizes?
5. On page 6, the authors state that int2MR may not be suitable when IVs are highly correlated. Could they clarify why and demonstrate, via simulations, the extent to which IV correlation affects the method’s performance?
6. Since the proposed method is Bayesian, it would be useful to assess how robust the proposed test statistics is against the misspecification of prior distributions. A discussion or sensitivity analysis for the simulated and particularly the real data would strengthen the conclusions.
